# Barriers to entry: A participatory science study of tick identification, monitoring, and physical barriers to reduce tick bites on horses

**DOI:** 10.1371/journal.pone.0357053

**Published:** 2026-10-07

**Authors:** Erika T. Machtinger, Karen C. Poh, Laura B. Kenny, Danielle N. Smarsh

**Affiliations:** 1 Penn State Extension, Pennsylvania State University, University Park, Pennsylvania, United States of America; 2 Department of Entomology, Pennsylvania State University, University Park, Pennsylvania, United States of America; 3 Penn State Extension, Pennsylvania State University, Collegeville, Pennsylvania, United States of America; 4 Department of Animal Science, Pennsylvania State University, University Park, Pennsylvania, United States of America; National Research Centre, EGYPT

## Abstract

Equine tick-borne diseases pose increasing challenges due to climate change and expanding tick geographical ranges. With no vaccines available for Lyme disease or equine anaplasmosis in horses, tick bite prevention remains the primary control method. This two-year study evaluated tick species composition on horses in Pennsylvania and assessed the effectiveness of physical barriers for tick prevention through community-based participatory research. The study engaged horse owners as research partners. Year 1 (2022) established baseline tick surveillance across 45 participants with 60 horses, conducting weekly tick checks from April to June. Year 2 (2023) evaluated physical barrier interventions using 50 horses assigned to three treatments: No Treatment (control), Physical Barrier (Untreated), and Physical Barrier (Treated with permethrin), with twice-weekly surveillance. A total of 413 ticks were collected, with species composition shifts between years. Species recovered included American dog ticks (*Dermacentor variabilis)*, blacklegged ticks (*Ixodes scapularis*), and longhorned ticks (*Haemaphysalis longicornis)*, which were detected for the first time in one of the counties in the study region. Tick attachment occurred across eight body locations and differed significantly between years. Physical barriers, particularly untreated fly boots and masks, were associated with reduced tick attachment. Comprehensive whole-body surveillance remains essential given consistent tick distribution across anatomical regions.

## Introduction

Equine tick-borne diseases pose growing challenges for horse caretakers across the United States. Climate change and evolving land-use patterns have expanded tick geographical ranges and increased disease incidence, creating new threats for horses in previously unaffected regions [1–4]. Currently, over 15% of equids and 29% of equine operations in the US report tick infestations, with blacklegged ticks (*Ixodes scapularis*) posing a particularly significant risk as a primary vector for *Borrelia burgdorferi* and *Anaplasma phagocytophilum*, the causative agents of Lyme disease and anaplasmosis, respectively [5]. Equine tick exposure can be influenced by landscape features. Many tick species prefer forested and edge habitats, so tick bite risk can increase near forested areas, woodland edges, and in settings where horses are ridden or turned out in wooded or transitional habitats, where tick densities tend to be greatest.

The geographic expansion of *I. scapularis* has led to increases in Lyme disease incidence and the extent of endemic areas over recent decades [6]. Longitudinal studies document this expansion through rising seroprevalence rates in affected regions. On a large Standardbred breeding operation in central New Jersey across two farm sites (>170 horses), seroprevalence increased from 12% among mares and 35% among yearlings in 1983 to 60% among mares and yearlings by 1985–1986 [7]. Similarly, equine anaplasmosis cases are increasing throughout the United States, with seroprevalence studies showing wide geographic variation in exposure to *A. phagocytophilum*, ranging from 66% in Texas to 100% in New York and New Jersey [8]. In a survey of 271 horses in Pennsylvania from 2017–2019, 44% tested positive for *A. phagocytophilum* and 77% tested positive for *B. burgdorferi* [9]. The clinical significance of these trends is reflected in owner experiences: a 2019–2020 Pennsylvania survey found that 28% and 16% of horse owners reported their animals had been diagnosed and treated for Lyme disease or equine anaplasmosis, respectively, with 67% of respondents expressing concern about tick-borne diseases (Poh, personal communication).

With no vaccines available for Lyme disease or equine anaplasmosis in horses, tick bite prevention remains the primary method for controlling these diseases. Current prevention approaches include topical acaricides, environmental modifications, and physical protective barriers. However, systematic documentation of the effectiveness of these strategies under varied management conditions remains limited, particularly for physical barrier methods. This knowledge gap limits evidence-based recommendations, given that physical barriers represent a promising approach that may reduce tick attachment while avoiding concerns about pesticide residues or the development of resistance.

Evidence from human studies demonstrates the potential of barrier protection for tick prevention. Permethrin-treated clothing has shown 73.6–100% protection against various tick species in human populations [10–12]. Miller et al. demonstrated that permethrin-impregnated clothing not only reduced the number of *I. scapularis* nymphs that attached to human volunteers but also caused the majority of attached nymphs to die within hours [13]. In equine applications, leg protection reduced stable fly defensive behaviors, suggesting that physical barriers may be both effective and well-tolerated [14]. Despite this promising evidence, no controlled evaluations of similar barrier efficacy exist for equine tick prevention.

Community-based participatory research provides a framework for evaluating prevention interventions under real-world equine management conditions while engaging horse owners as active research collaborators [15,16]. We implemented a two-year community-based study that engaged horse owners as research partners in both tick surveillance and the evaluation of tick prevention interventions. During Year 1, participants conducted systematic tick monitoring to develop proficiency with study protocols and characterize tick presence and attachment patterns on horses. During Year 2, participants implemented physical barrier interventions while continuing standardized tick surveillance, and only Year 2 data were used to evaluate intervention efficacy. Together, the two study phases generated information on tick species affecting horses in Pennsylvania, assessed the effectiveness of physical barriers for equine tick prevention, and demonstrated the feasibility of community-based research in equine health.

## Materials and methods

### Year 1. Evaluation of ticks on horses in Pennsylvania and development of owner confidence in tick monitoring practices

#### Study recruitment.

Participants were recruited using the Penn State Extension email list, press releases in local equine publications, and social media (Facebook, Meta Platforms Inc., Menlo Park, CA) from January to March 2022. Prospective participants completed an online survey (Microsoft Forms, Redmond, WA) to indicate interest. Participants were required to be located in Pennsylvania and be age 18 or older.

#### Tick check training and study preparation.

Prospective participants were asked to attend a webinar training session on the project and on properly performing a tick check on a horse. Two training sessions were offered on different days to accommodate schedules. Training videos demonstrated tick check procedures. A pre-project survey included three questions about specific details of the training to confirm engagement with training content. Participants who completed the training and survey were mailed a Penn State Tick Removal Kit for Horses containing tweezers, four small, zippered plastic bags for tick storage, a marker, a small magnifying glass, a tick identification and information card for horses, and five tick collection and testing cards. During the seven-week study period, participants were required to: (1) perform twice-weekly tick surveillance examinations following the standardized training protocols presented in the training, and (2) collect and submit any attached ticks to the research team.

#### Pre- and post-project surveys.

The pre-project survey (Qualtrics, Provo, UT) included questions about horse numbers, level of concern about ticks, and management practices for tick control (S1 File). A post-project survey was conducted after the data collection period to collect impact data from the project. Participants were asked to evaluate changes in their knowledge of tick topics and comfort levels with performing tick checks (S2 File). Participants were also asked to report changes in concern levels about tick bites and their intentions to continue performing tick checks and to adopt new tick control practices after the study.

### Year 2. Evaluation of physical barriers to reduce tick bites on horses

#### Study recruitment.

In contrast to Year 1, which used broad inclusion criteria for surveillance and training, Year 2 implemented more restrictive inclusion and exclusion criteria to support evaluation of a controlled intervention. These included requirements for prior tick exposure, standardized horse size, and restrictions on concurrent tick control product use to minimize confounding effects. Participants were recruited through Penn State Extension email distribution lists and social media platforms (Facebook, Meta Platforms Inc., Menlo Park, CA) from November 2022 until February 2023. Individuals who participated in the previous tick surveillance study were also invited to enroll in the current investigation. An online survey administered via the Qualtrics platform was used to collect applications and facilitate participant selection.

Participants were enrolled based on the following inclusion criteria: age ≥ 18 years, ownership of at least one horse standing ≥15.0 hands in height (to standardize physical barrier sizing), and documentation of at least one tick attachment on their horse(s) within the 12 months preceding enrollment. During the four-week study period, participants were required to: (1) outfit enrolled horses with research team-provided fly boots and masks during turnout periods, (2) perform twice-weekly tick surveillance examinations following standardized training protocols, (3) collect and submit any attached ticks to the research team, and (4) refrain from using additional tick or fly control products during the study period.

Enrolled participants completed a pre-project survey administered via the Qualtrics platform (S3 File). The survey instrument collected demographic data on enrolled equines and product specifications, confirmed participant adherence to study protocols, and included identical tick management practice questions from the Year 1 assessment to ensure longitudinal consistency in data collection.

Enrolled participants completed an online tick check training hosted by the Penn State Extension Equine Team. This training covered the background and purpose of the study, the data being collected, the study methods and protocol, and how to apply the physical barriers.

#### Treatments.

Horses were assigned to one of three treatments: 1) a No Treatment (Control) group where horses were evaluated with no protective measures against ticks or other ectoparasites; 2) a Physical Barrier (Treated) group where horses were outfitted with Shoo Fly Leggins fly boots (Stone Manufacturing & Supply Co., Kansas City, Missouri) and all round mesh fly mask (Harrison Howard Equestrian, England) treated with Sawyer’s permethrin (Sawyer Products, Inc., Safety Harbor, FL) according to the clothing label description and allowed to dry; 3) and a Physical Barrier (Untreated) group where horses outfitted with the same fly boots and fly mask but with no permethrin treatment. Participants provided fly boots and mask sizing to the research team for treatment and distribution to each horse. Participants were provided with four fly boots and one fly mask if they had animals in the treatment groups. Fly boots and masks were labeled with the horse’s name and mailed to participants. Participants enrolled in the physical barriers groups were instructed to have horses wear fly boots and fly masks whenever horses were turned out, but not when riding, and to record whether physical barriers were removed or lost.

Treatments were semi-randomly assigned. For owners with three horses, each horse was given one of the three treatments at random. Those with one or two horses were randomly assigned to treatment, ensuring that each treatment group had the same number of horses. The owners were blinded to whether their animals were in the Physical Barrier (Treated) or Physical Barrier (Untreated) groups. All participants were provided with tick collection bags, labeled return poly mailers, and printed data sheets. Participants could also provide data electronically through a Qualtrics platform.

#### Tick checks and identification.

Participants were instructed to conduct full-body tick checks twice weekly from April 18 to June 5, 2022, for Year 1, and from April 3 to April 30, 2023, in Year 2, to focus on *I. scapularis* phenology, with at least 48 hours between checks. Participants were provided with paper data collection sheets and an online data collection survey (Qualtrics). Both options included the same fields, and participants could choose either. Data collected included name, date of check, horse name, study week, horse activity in the past 24h, number of ticks found, and location of ticks found. In both years, participants recorded the body location where ticks were found (body, crest/mane, between front legs, front legs, head, between hind legs, hind legs, tail). Participants were asked to return ticks for identification. Ticks sent back were identified to species and life stage using published morphological characters [17,18], except for *Haemaphysalis longicornis* specimens, which were confirmed by the United States Department of Agriculture’s National Veterinary Service Laboratory (Ames, IA).

This study was conducted in accordance with Penn State Institutional Animal Care and Use Committee Protocol PROTO202202182. This study was reviewed by the Institutional Review Board under protocol STUDY00016457 and was determined to be exempt from IRB oversight.

### Statistical analysis

#### Pre-and post-surveys.

Descriptive statistics from all surveys are presented as a percentage of the number of respondents for each question. Since not all respondents answered each question, the number of respondents is also presented. When multiple selections were allowed, the data presented are the percentage of respondents who chose that selection. Duplicate entries were identified by name, and when individuals submitted multiple responses, the most complete submission was retained.

#### Tick species.

Tick species composition data were analyzed to compare relative abundance and distribution patterns between Year 1 (April 18 – June 5, 2022) and Year 2 (April 3 – April 30, 2023). Chi-square goodness of fit tests were performed to assess whether the observed species distribution in Year 2 differed significantly from that expected based on Year 1 proportions. Percent changes in tick species abundance were calculated as (Year 2 count – Year 1 count)/Year 1 count × 100. For species absent in Year 1 but present in Year 2, changes were reported as “new species.” Because sampling periods differed between years, percent changes were used only as directional comparisons relative to the Year 1 baseline and should not be interpreted as standardized estimates of interannual change. Differences in species abundance may reflect seasonal tick phenology associated with the shorter, earlier sampling period in Year 2, as well as differences in sampling locations, habitat characteristics, and local ecological conditions between years. Simpson’s diversity index was calculated to assess community evenness between years using the formula $D=\text{1}-\Sigma \left({\text{pi}}^{\text{2}}\right),$ where pi represents the proportion of each species. All statistical analyses were performed using standard chi-square procedures.

#### Tick attachment sites.

Because several attachment-site categories had small counts or zero observations, differences in anatomical distribution between years were evaluated using Fisher’s exact test with Monte Carlo simulation. All comparisons were conducted using adjusted Year 1 counts, in which ticks collected from a single outlier horse (Z-score = 4.19) were removed, and observed counts from Year 2. To further assess differences at individual body locations, post hoc comparisons were conducted using Fisher’s exact tests for each anatomical region. P-values from these tests were adjusted for multiple comparisons using the Benjamini–Hochberg procedure. Percent changes were calculated as (Year 2 − Year 1)/Year 1 × 100.

#### Physical barrier treatment.

We combined multiple tick observations per horse-week-treatment, creating 224 analytical units from 445 individual assessments across 50 horses in the Year 2 study. To calculate the weekly prevalence of ticks, the number of observations with at least one tick observed was divided by the total number of observations for that week.

We analyzed tick data as presence/absence rather than counts, coding observations as tick present (1) or absent (0). We used a generalized linear mixed model (GLMM) with a binomial distribution and logit link to account for the binary nature of the response and repeated measures across Pennsylvania horses. When physical barriers were misused, missing, or removed, we recoded those observations as “No Treatment” to maintain analytical integrity.

Three candidate models were fit using the glmmTMB package in R v. 4.4.x to account for the high frequency of zero observations (79% of observations) [19]: (1) a binomial GLMM with no zero-inflation, (2) a ZIGLMM with intercept-only zero-inflation, and (3) a ZIGLMM with treatment-specific zero-inflation. Model comparison using Akaike Information Criterion (AIC) calculated via the stats package revealed that the standard GLMM provided equivalent fit to zero-inflated alternatives. Additionally, likelihood ratio tests implemented through the anova() function confirmed that treatment did not significantly affect the probability of structural zeros (χ^2^ = 1.30, p = 0.522), further supporting the use of the binomial GLMM.

The final GLMM included fixed effects for treatment group (No Treatment, Physical Barrier Treated, Physical Barrier Untreated) and week (continuous), with random intercepts for individual horses to account for repeated measures and animal-specific variation in tick susceptibility. We used No Treatment as the reference category for treatment comparisons, specified using the relevel() function. The model was fit using maximum likelihood estimation with Laplace approximation in glmmTMB. We assessed model adequacy using simulation-based scaled residual diagnostics from the DHARMa package, which generate standardized residuals appropriate for mixed models with non-normal responses. Model fit was further evaluated by comparing observed versus predicted zero frequencies using the predict() function.

We evaluated practical effectiveness using measures for binary outcomes recommended by Cook and Sackett and Laupacis et al. [20,21]. We calculated absolute risk reduction (ARR) as the difference between the control and treatment rates, and the number needed to treat (NNT) as the reciprocal of ARR. We derived odds ratios with 95% confidence intervals from model parameters. Statistical significance was assessed at α = 0.05. Clinical interpretation focused on baseline risk, intervention costs, and practical implementation rather than standard, but arbitrary, statistical thresholds.

## Results

### Demographic data from pre- and post-project surveys

In Year 1, 45 people completed pre-project surveys, and 33 people returned at least one data sheet (S4 File). They represent 21 Pennsylvania counties. Only 22 people (66.7%) returned at least one data sheet each week of the study. In Year 2, 51 people completed pre-project surveys, and 21 people returned at least one data sheet, representing 19 counties. Sixteen people (76%) returned the proper number of complete and correct data sheets for each horse. Eight participants completed both studies.

### Year 1 surveys

Fifty-one people started the pre-project survey, and 45 completed it. The first three questions were to confirm that participants understood the requirements of the study. Correct answers were chosen by 95.7, 97.8, and 84.8% of participants, respectively (n = 46). Most respondents (39.1%; n = 46) owned two horses, followed by 32.6% owning three to five horses. Half of respondents were “very concerned” about ticks on their horses (50.0%; n = 46), while 43.5% were “somewhat concerned” and 6.5% were “neutral.” During the tick transmission season between April and November, 50.0% (n = 46) of respondents kept horses on pasture for 16 or more hours daily. Most respondents (84.8%; n = 46) turn their horses out near or in forested habitat or forest edges.

Before this study, 47.8% (n = 46) performed full-body tick checks on horses only when they remembered or when they found a tick. Forty-three percent performed full-body tick checks weekly or more frequently. However, when asked about partial-body tick checks, 75.6% (n = 45) reported performing them weekly or more often. The top challenges to conducting tick checks were finding ticks in the coat (51.1%, n = 45), remembering to do them (46.7%), the time required to complete them (28.9%), and knowing where to look (11.1%). Most respondents check their horses for ticks during spring (82.6%; n = 46), summer (87.0%), and fall (84.8%), while 15.2% continue to check throughout the winter. Fifty-nine percent (n = 46) have observed one horse getting more ticks than others in the same pasture. When respondents were asked to report the number of times per year they found ticks on their most tick-prone horse, 32.6% (n = 46) selected 6–10 times per year, and 30.4% selected 11 or more times per year. When asked how many ticks they found on their most tick-prone horse on each occasion, 71.7% selected 1–2 ticks per occasion. Respondents were asked to click on one area of a horse illustration to represent where they find the most ticks on their horses. The most common areas selected were under the jaw and on the chest (Fig 1A; n = 45).

**Fig 1:**
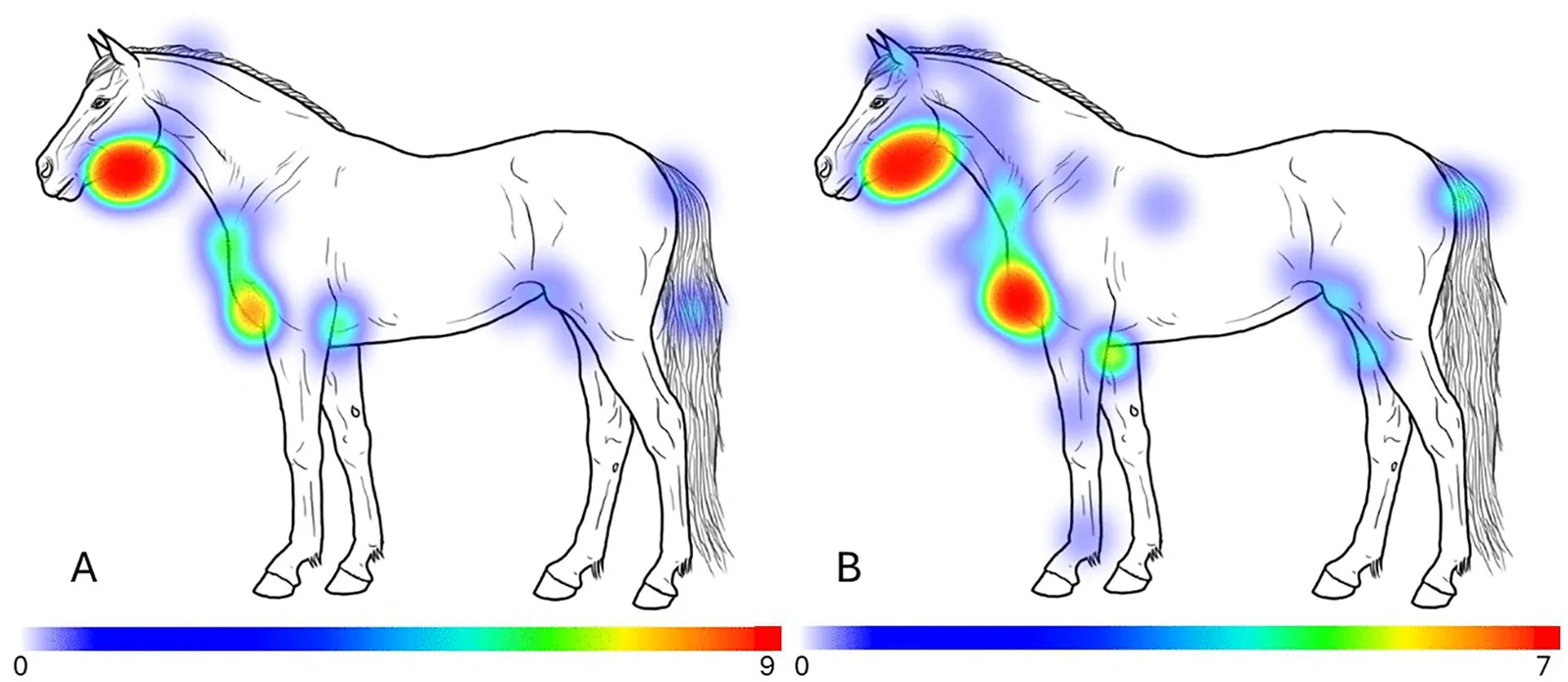
A) Areas of the horse where respondents of the Year 1 pre-survey reported finding the most ticks on horses in their care. Red areas indicate that more respondents selected that area, while blue areas indicate fewer selections (n = 45). B) Areas of the horse where respondents of the Year 2 pre-survey reported finding the most ticks on horses in their care (n = 45). Red areas indicate that more respondents selected that area, while blue areas indicate fewer selections.

When asked to select all tick control methods used on horses in their care, respondents mainly selected on-animal control (i.e., repellents) (93.5%; n = 46) and pasture or landscape management (i.e., trimming vegetation, moving fence lines away from wooded areas, mowing, etc.) (71.4%). Very few respondents used landscape chemicals (i.e., spraying chemicals in areas where ticks might be) (6.5%), and 2.1% didn’t use any tick control methods. Respondents were then asked to select their primary method of tick control, and the most frequently used method was on-animal control (65.2%; n = 46), followed by pasture or landscape management (23.9%). Finally, respondents selected which on-animal repellent products they used specifically to control ticks. Most used synthetic spray repellants, i.e., permethrin (45.7%; n = 46), followed by Equispot (32.6%), natural spray repellents, i.e., Outsmart, Ecovet, Equiderma, etc. (32.6%), homemade repellent (15.2%), and fipronil (2.2%). Additionally, 26.1% reported using no products specifically to prevent tick bites.

Thirty-six participants completed the post-project survey. Knowledge gain was rated for three topics on a scale of 1–5, with 1 being no knowledge gain and 5 being a great deal of knowledge gain. Respondents reported a mean knowledge gain of 3.53 for general tick information (life cycles, habitat, identification); 3.14 for tick-borne diseases in horses; and 3.72 for how to conduct a tick check on their horse. Increased confidence in performing tick checks on horses was rated on the same 1–5 scale, with 1 indicating no increase in confidence at all and 5 indicating a great deal, yielding a mean score of 3.81. Common challenges in conducting tick checks included finding ticks on the coat (33.3%), remembering to do them (30.6%), and the time required to complete them (27.8%). Most respondents (86.1%) report that they intended to continue performing regular full-body tick checks on some or all their horses after the completion of the study. Most respondents did not change any tick control practices during the study (77.8%). Those who made changes reported switching to fly spray products containing tick repellents, increasing their tick checks, and spot-spraying the horse’s areas where ticks were found. Few respondents said they intend to adopt new practices for their horse or farm as a result of the study (25%). New practices reported by respondents include more frequent tick checks, changing fly-spray products, mowing borders around pastures, and testing ticks for tick-borne diseases. Most respondents (94.4%) did not intend to discontinue any tick control methods as a result of the study. General concern about the risk of tick bites on horses remained unchanged for 69.4% of respondents, while 22.2% became a little or much more concerned. During the study, 66.7% of respondents found fewer ticks than expected on their horses, while only 11.1% found more. Many people commented that they had seen many more ticks earlier in the spring and the previous year than during the study period.

### Year 2 survey

Fifty-three people began the pre-project survey, and 51 completed it. Most respondents owned three to five horses (50.9%; n = 53), and 41.5% owned more than six horses. Most respondents reported finding the most ticks on their horses during the fall months (45.3%; n = 53), followed by summer months (34.0%) and spring months (20.8%). Seventy percent of respondents were “very concerned” about finding ticks on horses (n = 53), with an additional 30.2% “somewhat concerned.” Most respondents (60.4%; n = 53) kept their horses on pasture for 16 hours or more per day during the tick transmission season of April through November. Eighty-nine percent of respondents turned horses out on or in forested habitat or along forest edges (n = 53). Before this study, most respondents (52.8%; n = 53) conducted full-body tick checks weekly, and 26.4% did so only when they remembered or found a tick. Partial-body tick checks were conducted daily by 48.1% of respondents (n = 52) and weekly by 28.9%. The most common challenges in conducting tick checks were finding ticks in the coat (53.9%; n = 52), the time required to complete them (32.7%), and remembering to do them (30.8%). Respondents reported checking their horses for ticks during the spring (77.4%; n = 53), summer (81.1%), and fall (88.7%), with 22.6% checking during the winter. Most respondents (69.8%; n = 53) have observed some horses getting more ticks than others in the same pasture.

When asked about the horse that gets the most ticks, respondents reported finding ticks on this horse 6–10 times per year (30.2%; n = 53) or 11 or more times per year (34.0%). Most typically found 1–2 ticks per occurrence (64.1%; n = 53) on the horse that gets the most ticks. Respondents were asked to click on one area of a horse illustration to represent where they find the most ticks on their horses. The most common regions selected were under the jaw and on the chest (Fig 1B; n = 45).

When asked to select all tick control methods used on the farm, the most commonly used method was on-animal control (88.7%; n = 53), followed by pasture or landscape management (54.7%). Similarly, the most frequently used tick control method was on-animal control (73.7%; n = 5), followed by pasture or landscape management (15.4%). Products explicitly used to control ticks included synthetic spray repellents (53.9%; n = 52), Equispot (34.6%), natural spray repellents (30.8%), homemade repellents (17.3%), and fipronil (5.8%). Fifteen percent used no products specifically for tick control.

### Tick species composition and abundance

A total of 413 ticks were collected across both study periods, with 332 ticks (n = 60 horses) in Year 1 (2022; adjusted to 46 ticks following removal of 286 ticks from one outlier animal) and 81 ticks (n = 50 horses) in Year 2 (2023), representing a 75.6% decline in overall abundance based on adjusted totals (Table 1). Of the total ticks, 230 were returned for identification in Year 1 and 51 were returned in Year 2. All returned ticks were adults. Species composition differed markedly between years. *Dermacentor variabilis* decreased from 65.7% (n = 218) of identified ticks in Year 1 to 3.7% (n = 3) in Year 2 (−98.6%), whereas *Ixodes scapularis* increased from 3.6% (n = 12) to 51.9% (n = 42) (+250.0%). *Haemaphysalis longicornis* was not detected in Year 1 but was identified in Year 2 (2.5%, n = 2). Specimens classified as unknown or not returned represented 30.7% (n = 102) of ticks in Year 1 and 42.0% (n = 34) in Year 2.

**Table 1:** Distribution and abundance changes of tick species removed from horses in Pennsylvania between Year 1 (April-June 2022) and Year 2 (April 2023).

Tick Species	Year 1 (April 18- June 5, 2022)		Year 2 (April 3-April 30, 2023)^a^		% Change (Y1 vs. Y2)^b^
	Ticks (N)	Ticks (% of total)	Ticks (N)	Ticks (% of total)	
*Dermacentor variabilis*	218	65.7%	3	3.7%	−98.6%
*Haemaphysalis longicornis*	0	0.0%	2	2.5%	New species
*Ixodes scapularis*	12	3.6%	42	51.9%	250.0%
Unknown/Not returned^c^	102	30.7%	34	42.0%	66.7%
Grand Total	332	100.0%	81	100.0%	75.6%

^a^The study time frame and tick check schedule were two checks weekly over seven weeks in year 1 (14 total tick checks) and two checks weekly over four weeks in year 2 (8 total tick checks).

^b^% Change = (Y2 − Y1)/Y1 × 100; shows the relative increase or decrease from Year 1 to Year 2 (directional, baseline = Year 1). Percent change values are based on counts from differing sampling periods (Year 1: April–June; Year 2: April only) and should be interpreted in the context of seasonal tick phenology.

^c^Some returned specimens were damaged (e.g., crushed or incomplete), limiting accurate identification. Additionally, not all participants submitted collected ticks, and species identification could therefore not be conducted for all observations.

Species composition differed significantly between years (χ² = 172.9, df = 3, *P* < 0.001); however, these differences should be interpreted in the context of differing sampling timeframes and seasonal tick phenology rather than as a direct year-to-year comparison. In Year 1 (April–June), *Dermacentor variabilis* was the dominant species, comprising 65.7% (n = 218) of all collected ticks, with most specimens (>90%) originating from a single horse. Unknown or not returned specimens accounted for 30.7% (n = 102), and *Ixodes scapularis* represented 3.6% (n = 12), with no *Haemaphysalis longicornis* detected. In contrast, during the more temporally restricted Year 2 sampling period, *Ixodes scapularis* was most abundant (51.9%, n = 42), followed by unknown specimens (42.0%, n = 34) and *Dermacentor variabilis* (3.7%, n = 3); *Haemaphysalis longicornis* was detected at low frequency (2.5%, n = 2).

### Tick attachment sites

Study participants found ticks on eight body locations (Fig 1). Tick attachment site distributions differed significantly between years (Fisher’s exact test with Monte Carlo simulation, *P* < 0.001), indicating a shift in the anatomical distribution of tick attachment. Descriptively, tick attachment increased across most body locations in Year 2 relative to Year 1 (Table 2), with the largest increases observed in the crest/mane (600.0%), body (233.3%), front legs (114.3%), and head (110.0%). In contrast, tick attachment decreased substantially in the front legs (between) region (−72.2%). The front legs (between) had the greatest proportion of ticks in Year 1 (35.3%) and this was the only statistically different location, whereas the head region had the highest proportion in Year 2 (25.9%). The head region consistently represented a substantial proportion of tick attachment in both years (19.6% in Year 1 and 25.9% in Year 2).

**Table 2:** Comparison of the anatomical distribution of tick attachment sites on Pennsylvania horses between Year 1 (April-June 2022) and Year 2 (April 2023).

Body Location	Year 1 (April 18- June 5, 2022)^2,3^			Year 2 (April 3-April 30, 2023)^b,c^		% Change (Y1 vs Y2)^c, d^
	Ticks (N)	Adjusted Ticks (N)^a^	Adjusted Ticks (% of total)^a^	Ticks (N)	Ticks (% of total)	
Body	32	3	5.9%	10	12.3%	233.3%
Crest/mane	4	2	3.9%	14	17.3%	600.0%
Front legs (between)	19	18	35.3%	5	6.2%	−72.2%*
Front legs	8	7	13.7%	15	18.5%	114.3%
Head	59	10	19.6%	21	25.9%	110.0%
Hind legs	6	5	9.8%	7	8.6%	40.0%
Hind legs (between)	15	6	11.8%	7	8.6%	16.7%
Tail	189	0	0.0%	2	2.5%	NA
TOTALS	332	51	100.0%	81	100.0%	58.8%

^a^“Adjusted ticks” removes 286 ticks collected from one individual that was identified as an extreme statistical outlier.

^b^The study time frame and tick check schedule were two checks weekly over seven weeks in Year 1 (14 total tick checks) and two checks weekly over four weeks in Year 2 (8 total tick checks).

^c^The total number of horses with ticks in Year 1 was 21 out of 60, and in Year 2 was 21 out of 50.

^d^% Change = (Y2 − Y1)/Y1 × 100; shows the relative increase or decrease from Year 1 to Year 2 (directional, baseline = Year 1).

*Indicates statistically significant difference between years for that body location based on Fisher’s exact test (P < 0.05), with Benjamini–Hochberg correction for multiple comparisons.

### Treatment effects

Tick checks in April 2023 resulted in a total of 224 tick observations. Most observations (79.0%) showed no ticks present. Each horse contributed 1–8 observations (median = 3). Horses were distributed across three treatment groups: No Treatment (102 observations, 45.5%), Physical Barrier (Treated) (53 observations, 23.7%), and Physical Barrier (Untreated) (69 observations, 30.8%).

The overall tick encounter rate varied significantly across treatments, ranging from 13.0% in the Physical Barrier (Untreated) group to 28.4% in the No Treatment group (Table 3). Approximately 54% of horses (27/50) did not have ticks during the study. Individual horses varied considerably in their baseline susceptibility to ticks (σ² = 3.01) (Table 4). None of the treatments were statistically significant, so practical or clinical benefits were evaluated. The Physical Barrier (Untreated) had a protective effect (OR = 0.38), with a 62% reduction in tick encounter risk. Physical Barrier (Treated) showed tick-encounter protection, with a 51% reduction in tick encounters (OR = 0.49) (Table 4). Physical Barrier (Untreated) demonstrated protective efficacy, with a number needed to treat (NNT) of 6.5, indicating that 6.5 horses would need to be treated with untreated barriers to prevent one tick encounter, compared with 8.7 for Physical Barrier (Treated) (Table 3). Combined with the reduction of the odds of tick encounter (Table 4) compared to No Treatment, Physical Barrier (Untreated) qualified as providing practical benefit, given its clinically meaningful effect size. Physical Barrier (Treated) was classified as providing an uncertain benefit, given a modest effect size and a confidence interval that included the null value.

**Table 3:** Effect sizes and clinical significance for Pennsylvania horses during a physical barrier treatment study to reduce tick bites in April 2024.

Treatment Group^a^	Tick Checks (n)	Horses Checked (n)	Tick Present n (%)	Tick Absent n (%)	Event Rate % (95% CI)^b^	ARR (95% CI)^c^	NNT^d^	Odds Ratio (95% CI)^e^	Practical Interpretation^f^
No Treatment	102	37	29 (28.4)	73 (71.6)	28.4 (20.2, 38.2)	Reference	—	1.00 (Reference)	Reference group
Physical Barrier (Treated)	53	15	9 (17.0)	44 (83.0)	17.0 (9.2, 29.2)	11.5 (−1.9, 24.8)	8.7	0.51 (0.22, 1.19)	Effect uncertain
Physical Barrier (Untreated)	69	19	9 (13.0)	60 (87.0)	13.0 (7.0, 23.0)	15.4 (3.6, 27.2)	6.5	0.38 (0.17, 0.86)	Practical benefit
**Total**	224	50	47 (21.0)	177 (79.0)	21.0 (16.0, 26.8)	—	—	—	—

^a^Treatment descriptions include No Treatment (Control) = Horses evaluated with no protective measures against ticks or other ectoparasites, Physical Barrier (Treated) = Horses protected against ticks with fly boots and fly mask treated according to clothing treatment direction with fabric-binding permethrin, and Physical Barrier (Untreated) = Horses protected against ticks with fly boots and fly mask with no permethrin treatment.

^b^Event rate is defined as the proportion of observations with tick presence within each treatment group, calculated as (number with ticks / total observations) × 100, with 95% confidence intervals.

^c^Absolute risk reduction (ARR) calculated as the difference between the control event rate and the treatment event rate, calculated as ARR = Event Rate_(no treatment)_ - Event Rate_(treatment)_. Positive values indicate benefit from treatment (Cook and Sackett 1995, Laupacis et al. 1988).

^d^The number needed to treat (NNT) represents the number of horses that must receive treatment to prevent one additional tick infestation compared to no treatment, calculated as NNT = 1/ARR (when ARR > 0). Lower values indicate more efficient intervention (Cook and Sackett 1995, Laupacis et al. 1988).

^e^Odds ratio is the ratio of odds of tick presence in the treatment group versus the control group, calculated as OR = (aᵢ × d₀)/(bᵢ × c₀), where aᵢ = ticks present in treatment group, bᵢ = ticks absent in treatment group, c₀ = ticks present in control, d₀ = ticks absent in control. Values <1.0 indicate a protective effect; values >1.0 indicate an increased risk.

^f^Practical interpretations are defined as *Practical benefit*: Clinically meaningful effect size (ARR ≥ 15% or NNT ≤ 7) with statistical significance (95% CI for OR excludes 1.0) and *Effect uncertain*: The estimated effect did not meet the prespecified threshold for clinical importance (ARR < 15% and NNT > 7), or the confidence intervals indicated statistical imprecision by including 1.0 for the OR or 0 for the ARR.

**Table 4:** Fixed effects from generalized linear mixed model predicting tick presence in Pennsylvania Horses.

Parameter^1^	Treatment Group	Coefficient (β)	SE^a^	Odds Ratio	95% CI^b^	p-value	Interpretation
Intercept	No Treatment	−1.646	0.65	0.193	(0.054, 0.688)	0.011	Baseline odds
Treatment	Physical Barrier (Treated)	−0.722	0.632	0.486	(0.141, 1.675)	0.253	51.4% odds reduction
Treatment	Physical Barrier (Untreated)	−0.967	0.633	0.380	(0.11, 1.316)	0.127	62% odds reduction
Week (per week increase)	—	0.029	0.182	1.029	(0.72, 1.47)	0.875	2.9% odds increase per week

^a^Individual horse intercepts showed substantial between-horse variation in baseline tick susceptibility (σ^2^ = 3.01, 95% CI: 0.95–8.8).

^b^SE = Standard Error; CI = Confidence Interval

There was moderate week-to-week variation in tick activity during the study period. Week 3 had the highest tick encounters (29.3%), while Week 2 had the lowest (15.8%). This 13.5 percentage point range suggests moderate temporal variation in tick activity across the sampling periods, with no clear seasonal trend (Table 5). The variation may reflect natural fluctuations in tick activity or environmental conditions.

**Table 5:** Weekly observations of ticks on Pennsylvania horses during a physical barrier treatment study to reduce tick bites in April 2023.

Study Week^a^	Total Observations (n)	Individual Horse Observations (n)^b^	Observations with Tick Presence n (%)	Observations with Tick Absence n (%)
Week 1	59	49	13 (22.0)	46 (78.0)
Week 2	57	50	9 (15.8)	48 (84.2)
Week 3	58	49	17 (29.3)	41 (70.7)
Week 4	50	45	8 (16.0)	42 (84.0)
**Total**	**224**	**193**	**47 (21.0)**	**177 (79.0)**

^a^Participants conducted two tick checks per week during the study, which were combined for the overall week total tick presence and the total observations that resulted in at least one tick found on a horse.

^b^Individual horse observations are the total number of individual horses that were observed to have at least one tick.

## Discussion

This two-year investigation demonstrates the value of participatory science for advancing understanding of equine tick management. By engaging horse owners directly in surveillance and intervention trials, we generated regionally relevant data on tick burden while simultaneously building participant knowledge and confidence in monitoring practices. The approach revealed differences between study years, with overall tick encounters increasing by over 50% and a complete reversal in dominant species, from *Dermacentor variabilis* in 2022 to *Ixodes scapularis* in 2023. Sampling was conducted over seven weeks from April 18 to June 5 in 2022 and four weeks from April 3 to April 30 in 2023. Importantly, the untreated physical barriers showed measurable reductions in tick encounters under real-world conditions, underscoring their potential as a low-cost, practical management tool. Together, these findings illustrate not only the dynamic and shifting nature of equine tick exposure in the northeastern United States but also the power of community-based research to generate actionable evidence for both veterinary and public health contexts.

Pennsylvania horse owners have been asked in the past about tick concerns and tick control on their horses. In the current study, in Year 1, 50% of respondents reported being very concerned about finding ticks on their horses, but in Year 2, 70% were very concerned. In a 2021 survey of PA horse owners, approximately 70% of respondents reported being extremely or very concerned about their horses contracting a tick-borne disease [22]. The lower level of concern reported in Year 1 is unclear but may reflect differences in participants’ prior experience with tick-borne disease or the substantially smaller sample sizes in the current study. Year 1 and Year 2 reported finding 1–2 ticks on horses most frequently, indicating that, based on their reporting, the tick load was similar. Additionally, the risk of tick bites due to turnout in or near forested areas was similar between years (85% in Year 1 and 89% in Year 2).

Nearly half (43%) of Year 1 respondents reported conducting full-body tick checks on their horses weekly, and a similar proportion (53%) in Year 2 reported doing so weekly. A 2021 survey found that 41% of Pennsylvania horse owners performed weekly full-body tick checks [22]. Based on these numbers, it appears that in recent years, there has been no drastic change in the number of Pennsylvania horse owners performing full-body tick checks on their horses weekly. Tick checks are important for preventing pathogen transmission in humans [23]; thus, further education of horse owners is needed to emphasize the importance of regular full-body tick checks.

Consistently, the primary method of tick control reported was on-animal control (65% in Year 1 and 89% in Year 2). The 2021 survey found 60% of Pennsylvania horse owners used on-animal control as their primary method of tick control. It appears from these three surveys that the use of on-animal control for Pennsylvania horses is increasing over time; however, these results may be biased, as our study population was more informed and engaged than the general population of equine caretakers. While on-animal control can be useful for controlling ticks, its effects are typically short-lived and weather-dependent [24].

In both years, participants were asked at the start of the study where they typically find ticks on their horses. The most frequently chosen locations in both years were under the jaw and on the chest. In Year 1, the most frequent locations of ticks were between the hind legs, between the front legs, and on the head. Participants underestimated the number of ticks found between the hind legs, perhaps because this area is more challenging to see and access during routine grooming. In Year 2, the most frequent tick locations were the head and the front legs.

The program’s impact on Year 1 participants was assessed via an online follow-up survey. The program was effective at increasing self-assessed knowledge and comfort level with tick management. Behavior change was minimal, as this cohort reported that they had already been checking horses for ticks frequently before the project, suggesting a high level of concern about tick bites. Three respondents reported checking for ticks daily, and nine checked 2–6 times per week. Five indicated that they plan to check daily after the project, and twelve will check 2–6 times per week. Participants may have expected to collect more ticks than they ultimately found, and the lower-than-expected tick recovery may have contributed to this behavioral change by creating a false sense of security regarding the likelihood of tick bites.

This two-year study of tick parasitism on horses in Pennsylvania revealed dramatic temporal shifts in species composition, substantial overall abundance declines, and promising evidence for physical barrier protection strategies. The tick community composition reversed completely between years, with *D. variabilis* dominating collections in 2022 while *I. scapularis* was most abundant in 2023. Unadjusted tick numbers dropped significantly in the second year, and we found the invasive *H. longicornis* in a new Pennsylvania county (Clinton). Physical barriers worn by the horse reduced tick encounters compared with no treatment; treated and untreated barriers showed evidence of protection (Table 3), although differences between barrier types were not statistically significant. The complete reversal in tick species dominance between study years reflects not only location and microecology differences, but likely primarily phenological differences rather than ecological succession, as the sampling periods targeted seasonal activity windows for different tick species. The extended 2022 sampling period encompassed the peak spring activity of *D. variabilis*, which typically exhibits bimodal activity with spring peaks in May-June in northeastern regions [25,26]. The shorter 2023 sampling window aligned with the approximate time when overwintered *I. scapularis* adults emerge in early spring [27]. The phenological separation observed aligns with broader patterns among northeastern tick communities documented in wildlife studies [27], suggesting that effective protection strategies must account for season-long exposure risks rather than focus on single-peak periods. In addition, the extreme number of *D. variabilis* on a single horse in 2022 highlights the importance of individual-animal and environmental factors in determining heterogeneity in tick burden, a pattern consistent with observations in other livestock systems [28].

Year-to-year variation in tick abundance is likely driven by environmental conditions that influence tick survival and activity. Winter severity and early spring temperature and precipitation patterns can strongly affect overwintering success and subsequent questing behavior [29,30]. Differences in sampling design between years may also have contributed to the observed variation, as the 2023 sampling period targeted adult blacklegged ticks, potentially increasing their representation in collections. However, the majority of ticks recovered in unadjusted counts were American dog ticks, suggesting that species composition and sampling emphasis influenced observed patterns. The detection of *H. longicornis* in 2023 collections, although numerically small and documented in other Pennsylvania counties, was the first detection in Clinton County, Pennsylvania, and highlights the rapid range expansion of this invasive species throughout the eastern United States since its initial detection in New Jersey in 2017 [31,32]. This parthenogenetic tick feeds on large ungulates and, although it has not been associated with transmission of equine pathogens, heavy infestations have caused blood loss, anemia, reduced productivity, and mortality in cattle, highlighting the potential impact of high tick burdens on large animal hosts [33].

Excluding the difference in tick attachment between the front legs from Year 1 to Year 2, tick distributions across anatomical regions were generally similar between years, despite differences in overall abundance. This pattern suggests that attachment-site use may be relatively consistent across conditions, although some variation was observed. These findings contrast with studies suggesting species-specific attachment preferences based on morphological differences [34], but the patterns observed here were not strong enough to clearly support or refute those relationships. Instead, the results are more broadly aligned with multi-species tick studies on wildlife hosts that report overlapping attachment patterns [35]. Given the observed variability and the limited sample size, additional research using larger datasets is needed to better evaluate potential species-specific attachment preferences. The relatively even distribution across body regions demonstrates that comprehensive whole-body tick checks are necessary for effective detection and removal, as focused inspection protocols could miss attachment sites. This supports recommendations from veterinary parasitology guidelines that emphasize thorough examination procedures [36]. These findings contribute valuable baseline data for an understudied system, as equine tick burden studies in the northeastern United States remain limited compared to the extensive literature on pet and wildlife hosts [37,38]. Future research should prioritize standardized sampling methodologies, enhanced specimen identification protocols, and adequately powered treatment trials to build upon these preliminary findings and develop evidence-based management recommendations adapted to regional ecological conditions.

Physical barrier treatments demonstrated practical benefits, although they lacked statistical significance, highlighting the distinction between statistical and practical significance in field studies with high natural variation. For the Physical Barrier (Untreated) vs. No Treatment, the number needed to treat was 6.5 for the Physical Barrier (Untreated), which indicates that approximately six horses require treatment to prevent one additional tick event. In practical terms, a farm managing 60 horses could expect to prevent approximately 10 tick bite events by implementing physical barriers, a meaningful population-level benefit given the potential for tick-borne disease transmission and associated veterinary costs. Conversely, the Physical Barrier (Treated) demonstrated insufficient reliability for clinical implementation. The fitted estimates indicated that both untreated and permethrin-treated barriers reduced blacklegged tick attachment. These findings suggest that physical exclusion alone may contribute to protection against blacklegged ticks, as has been reported for stable flies [14]. Several factors may explain the paradoxical treatment pattern, including permethrin degradation under field conditions, as documented in other fabric-treatment studies [39]; the inability of the permethrin product to bind to the material used in protective boots and masks; sampling bias; or sample size limitations. The high individual variation and abundance of zero-count observations created challenging analytical conditions, which likely contributed to the lack of statistical significance, which is a common issue in ectoparasite field studies that has led to recommendations for larger sample sizes and alternative analytical approaches [40].

Researchers conducting citizen science projects that rely on participant data must monitor attrition rates throughout the project. Of the 45 people selected for Year 1, only 33 participated, and 22 participated weekly as instructed (49% of the original group). In Year 2, 51 people were selected, 21 participated, and 16 (31% of the original group) followed the protocol exactly. Depending on the study design, incomplete participation may still be usable, but it is recommended to recruit significantly more participants than required to achieve statistical power.

## Conclusions

Based on these findings, practical management recommendations for equine tick control should emphasize comprehensive, season-long surveillance. Horse owners in the northeastern and midwestern United States should implement daily whole-body tick checks throughout the known phenology of regionally important ticks, paying particular attention to leg regions, head, and neck areas while recognizing that no single body region can be ignored. Physical barriers, such as fly boots and masks, should be considered protective measures. Veterinarians should collaborate with entomologists to develop season-specific communication strategies that highlight the seasonal tick and tick-borne pathogen exposure risks. Management efforts should focus on identifying and protecting high-risk individuals within herds, as individual variation appears to drive tick burden patterns more than temporal factors. Finally, the emergence of invasive *H. longicornis* necessitates continued surveillance and reporting to veterinary authorities, particularly given this species’ potential for rapid population expansion and novel pathogen transmission capabilities in North American contexts.

## Supporting information

S1 File Tick study pre-program survey used to assess participant characteristics, baseline tick-related practices, and study-protocol understanding. (DOCX)

S2 File Tick study post-survey used to assess participant experiences, perceived knowledge and confidence gains, and intended changes in tick-checking and tick-control practices. (DOCX)

S3 File Horse clothing project pre-survey used for participant screening and collection of baseline horse, tick-exposure, tick-management, and study eligibility information. (DOCX)

S4 File Pennsylvania county locations of project participants that returned datasheets from Years 1 and 2. (DOCX)
