# Supplementary material for: Barriers to entry: A participatory science study of tick identification, monitoring, and physical barriers to reduce tick bites on horses
Source: PLoS One. 2026 Oct 7;21(10):e0357053. doi: 10.1371/journal.pone.0357053 (PMC13645029; doi:10.1371/journal.pone.0357053)
Supplement: Supplementary file 1 [file pone.0357053.s001.docx]

Tick Study Pre-Program Survey

Start of Block: Default Question Block

Q1 First and last name:

________________________________________________________________

Q2 Are you 18 years or older?

- Yes  (1) 

- No  (2) 

Skip To: End of Survey If Are you 18 years or older? = No

Q3 Does your horse(s) reside in PA for the majority of the year?

- No  (1) 

- Yes  (2) 

Skip To: End of Survey If Does your horse(s) reside in PA for the majority of the year? = No

| Page Break |  |
| --- | --- |

Q4 The next 3 questions are from the training session. If you do not remember the answers, please review the recording.

Q5 This study is 7 weeks long. During each week, how many times per week will you report a tick check on at least one horse?

- 1  (1) 

- 2  (2) 

- 3  (3) 

- 4  (4) 

Q6 Preferably, how long should you wait between tick checks?

- 24 hours  (1) 

- 48 hours  (2) 

- 36 hours  (3) 

Q7 How long should you spend checking each side of the horse?

- 1-2 minutes per side  (1) 

- 3-5 minutes per side  (2) 

- 6-10 minutes per side  (3) 

- 11-15 minutes per side  (4) 

| Page Break |  |
| --- | --- |

Q8 The remaining questions are about your farm management. 

Q9 How many horses do you own or care for?

- 1  (1) 

- 2  (2) 

- 3-5  (3) 

- 6-10  (4) 

- 11+  (5) 

Q10 In which PA county do these horses reside for the majority of the year? 

________________________________________________________________

Q11 How concerned are you about ticks on your horses?

- Very concerned  (1) 

- Somewhat concerned  (2) 

- Neutral  (3) 

- Somewhat not concerned  (4) 

- Not concerned  (5) 

Q12 On average, from April through November, how many hours a day are the horses that you own or care for on pasture?

- <1 hr  (1) 

- 1-4 hrs  (2) 

- 5-8 hrs  (3) 

- 9-12 hrs  (4) 

- 13-16 hrs  (5) 

- 16+ hrs  (6) 

| Page Break |  |
| --- | --- |

Q13 Are any of your horses or those that you care for turned out near or in **forested habitat** or **forest edge** similar to these photos during the months of April-November?

  

- Yes  (1) 

- No  (2) 

- I don't know  (3) 

| Page Break |  |
| --- | --- |

Q14 Prior to this study, how often did you conduct full body checks for ticks on horses you own, ride, or care for from April-November?

- Daily  (1) 

- Weekly  (2) 

- Monthly  (3) 

- When I remember or find a tick  (4) 

- Never  (5) 

Q15 Prior to this study, how often did you conduct partial body checks for ticks on horses you own, ride, or care for from April-November?

- Daily  (1) 

- Weekly  (2) 

- Monthly  (3) 

- When I remember or find a tick  (4) 

- Never  (5) 

Q16 What do you find challenging about conducting tick checks? (Check all that apply)

- Finding ticks in the coat  (1) 

- Knowing where to look  (2) 

- The time it takes to complete  (3) 

- Remembering to do them  (4) 

- I've never conducted tick checks before  (5) 

Q17 Which season(s) do you typically check horses(s) you own, care for, or ride for ticks? (Select all that apply)

- Spring  (1) 

- Summer  (2) 

- Fall  (3) 

- Winter  (4) 

- I don't check for ticks  (5) 

| Page Break |  |
| --- | --- |

Q18 For multiple horses in the same pasture, have you observed some horses getting more ticks, or ticks more frequently, than other horses where they are pastured?

- Yes  (1) 

- No  (2) 

- Sometimes  (3) 

- I only have one horse  (4) 

Q19 Think about the horse you own, ride, or care for that gets the most ticks. On average, how many times per year do you find ticks on this horse?

- 1-2  (1) 

- 3-5  (2) 

- 6-10  (3) 

- 11+  (4) 

- I don't know  (5) 

Q20 Think about the horse you own, ride, or care for that gets the most ticks. When you do find ticks on this horse, how many ticks do you estimate you find on each occasion?

- 1-2  (1) 

- 3-5  (2) 

- 6-10  (3) 

- 11+  (4) 

- I don't know  (5) 

| Page Break |  |
| --- | --- |

Q21 Click on the **one** area of the horse that best represents where you find the **most** ticks on horses(s) you own, ride, or care for. 

| Page Break |  |
| --- | --- |

Q22 What methods of tick control do you use on your horse or horses in your care? (Check all that apply)

- Pasture or landscape management (i.e., trimming vegetation, moving fence lines away from wooded areas, mowing, etc.)  (1) 

- Landscape chemicals (i.e., spraying chemicals in areas ticks might be)  (2) 

- On-animal control (i.e., repellents)  (3) 

- Other  (4) 

- I do not use tick control methods on horses(s) I own, ride, or care for  (5) 

Q23 What is the primary method of tick control do you use on your horse or horses in your care? 

- Pasture or landscape management (i.e., trimming vegetation, moving fence lines away from wooded areas, mowing, etc.)  (1) 

- Landscape chemicals (i.e., spraying chemicals in areas ticks might be)  (2) 

- On-animal control (i.e., repellents)  (3) 

- Other  (4) 

- I do not use tick control methods on horses(s) I own, ride, or care for  (5) 

Q24 Do you use any of the following tick control methods or products to **specifically** control or prevent ticks from biting your horses (not for fly control)? (Select all that apply)

- Equispot  (1) 

- Spray (synthetic) repellents (i.e., permethrin)  (2) 

- Spray (natural) repellents (i.e., Outsmart, Ecovet, equiderma, etc.)  (3) 

- Fipronil  (4) 

- Homemade repellent  (5) 

- I don't use any products to specifically prevent tick bites  (6) 

End of Block: Default Question Block
