# Supplementary material for: Barriers to entry: A participatory science study of tick identification, monitoring, and physical barriers to reduce tick bites on horses
Source: PLoS One. 2026 Oct 7;21(10):e0357053. doi: 10.1371/journal.pone.0357053 (PMC13645029; doi:10.1371/journal.pone.0357053)
Supplement: Supplementary file 2 [file pone.0357053.s002.docx]

Tick Study Post-Survey

Start of Block: Default Question Block

Q1 Thank you for your participation in the Penn State tick study. Your responses are very important and will be used to develop new educational products for horse owners. 

In this final survey, we would like you to tell us your thoughts about participating in this study. Thank you.

Q2 Please rate how much your knowledge of the following topics increased as a result of participating in this study. 

|  | None at all (1) | A little (2) | A moderate amount (3) | A lot (4) | A great deal (5) |
| --- | --- | --- | --- | --- | --- |
| General tick information: life cycles, habitat, identification (1) |  |  |  |  |  |
| Tick-borne diseases in horses (2) |  |  |  |  |  |
| How to conduct a tick check on a horse (3) |  |  |  |  |  |

Q3 Please rate how much your confidence in performing tick checks on horses increased as a result of the project.

|  | None at all (1) | A little (2) | A moderate amount (3) | A lot (4) | A great deal (5) |
| --- | --- | --- | --- | --- | --- |
| Confidence performing tick checks (1) |  |  |  |  |  |

Q4 What did you find challenging about conducting tick checks? (Check all that apply)

- Finding ticks in the coat  (1) 

- Knowing where to look  (2) 

- The time it takes to complete  (3) 

- Remembering to do them  (4) 

- Other (please specify)  (5) __________________________________________________

Q5 How often did you perform tick checks on your horse(s) **before** participating in this study?

- Daily  (1) 

- 2-6 times a week  (2) 

- Once a week  (3) 

- Every few weeks  (4) 

- Once a month  (5) 

- When I remembered  (6) 

- When I found a tick  (7) 

- Never  (8) 

Q6 Will you continue to perform regular full body tick checks on all or some of your horses once the study is over?

- Yes, all of my horses (or I have one horse)  (1) 

- Yes, some of my horses  (2) 

- No, not regularly  (3) 

- No, never  (4) 

Display this question:

If Will you continue to perform regular full body tick checks on all or some of your horses once the... = Yes, all of my horses (or I have one horse)

Or Will you continue to perform regular full body tick checks on all or some of your horses once the... = Yes, some of my horses

Q7 How often do you plan to perform full body tick checks on your horse(s) after participating in the study?

- Daily  (1) 

- 2-6 times a week  (2) 

- Once a week  (3) 

- Every few weeks  (4) 

- Once a month  (5) 

Q8 Did you change any of your tick control practices **during** the study (i.e., start a new product or discontinue a practice)?

- Yes  (1) 

- No  (2) 

Display this question:

If Did you change any of your tick control practices during the study (i.e., start a new product or... = Yes

Q9 Please describe the change you made during the study.

________________________________________________________________

________________________________________________________________

________________________________________________________________

________________________________________________________________

________________________________________________________________

Q10 Do you plan to adopt any new tick control practices on your horse/farm as a result of this study?

- Yes  (1) 

- Yes but not because of the study  (2) 

- No  (3) 

- I'm not sure  (4) 

Display this question:

If Do you plan to adopt any new tick control practices on your horse/farm as a result of this study? = Yes

Q11 Please describe which methods you plan to adopt as a result of the study and why.

________________________________________________________________

________________________________________________________________

________________________________________________________________

________________________________________________________________

________________________________________________________________

Q12 Do you plan to discontinue any tick control methods on your horse/farm as a result of this study?

- Yes  (1) 

- Yes but not because of the study  (2) 

- No  (3) 

- I'm not sure  (4) 

Display this question:

If Do you plan to discontinue any tick control methods on your horse/farm as a result of this study? = Yes

Q13 Please describe which methods you plan to discontinue as a result of the study and why.

________________________________________________________________

________________________________________________________________

________________________________________________________________

________________________________________________________________

________________________________________________________________

Q14 Are you more concerned or less concerned about risk of tick bites on your horse(s) than you were before the study?

- Much more concerned  (1) 

- A little more concerned  (2) 

- About the same  (3) 

- A little less concerned  (4) 

- Much less concerned  (5) 

Q15 Please explain why your level of concern has increased, decreased, or stayed the same.

________________________________________________________________

________________________________________________________________

________________________________________________________________

________________________________________________________________

________________________________________________________________

Q16 Overall, did you find the number of ticks you expected on your horses?

- More ticks than expected  (1) 

- About what I expected  (2) 

- Fewer ticks than expected  (3) 

Q17 If you have any final comments about the study, please enter them here. If you have questions, please send the team an email.

________________________________________________________________

________________________________________________________________

________________________________________________________________

________________________________________________________________

________________________________________________________________

End of Block: Default Question Block
