# Supplementary material for: Barriers to entry: A participatory science study of tick identification, monitoring, and physical barriers to reduce tick bites on horses
Source: PLoS One. 2026 Oct 7;21(10):e0357053. doi: 10.1371/journal.pone.0357053 (PMC13645029; doi:10.1371/journal.pone.0357053)
Supplement: Supplementary file 3 [file pone.0357053.s003.docx]

Horse Clothing Project Pre Survey

Start of Block: Default Question Block

Q1 Thank you for your interest in our tick clothing study! We had so much interest in the project that we have a few more questions to ask before we select our project participants. The deadline to complete the survey is February 26. 

Q3 Are you 18 years or older?

- Yes  (1) 

- No  (2) 

Q2 Please fill out your name and shipping address where you would like the items shipped.

- Name  (1) __________________________________________________

- Address  (2) __________________________________________________

- Address 2  (3) __________________________________________________

- City  (4) __________________________________________________

- State  (5) __________________________________________________

- Postal code  (6) __________________________________________________

- Pennsylvania County  (7) __________________________________________________

Q6 How many horses do you plan to use in this study? 

We can use a maximum of 3 horses per participant. They must reside in PA. Each horse needs to wear a Medium (cob size), Large (horse size), or Extra Large (e.g., warmblood, draft) fly mask and a Small, Medium, or Large Shoofly legging to be eligible. (Shoofly measurements can be found here: http://shooflyleggins.com/product_info)

- 1  (1) 

- 2  (2) 

- 3  (3) 

Q12 Are you willing to do twice weekly full-body tick checks (we will train you; takes approx. 10 minutes per horse) on each horse in the study? You will need to complete a quick data sheet each time for each horse.

- Yes  (1) 

- No  (2) 

Q13 Are you willing to **NOT** apply any additional tick or fly control methods during the study (4 weeks starting in early April)?

- Yes  (1) 

- No  (2) 

Q14 Participants will be required to attend one virtual training session before the study begins. Please indicate which training(s) you could attend.

- March 13, 2023 7pm  (1) 

- March 15, 2023 7pm  (2) 

- Neither of these  (3) 

Q5 During which season do you typically find the most ticks on your horses?

- Winter (Dec-Feb)  (1) 

- Spring (Mar-May)  (2) 

- Summer (Jun-Aug)  (3) 

- Fall (Sep-Nov)  (4) 

Q10 If you are selected to participate in the spring, would you be willing to do this study again in the fall (September to October) of this year? 

- Yes  (1) 

- Maybe  (2) 

- No  (3) 

End of Block: Default Question Block

Start of Block: Repeated Questions from Other Project

Q9 How many total horses do you own or care for?

- 1  (1) 

- 2  (2) 

- 3-5  (3) 

- 6-10  (4) 

- 11+  (5) 

Q11 How concerned are you about ticks on your horses?

- Very concerned  (1) 

- Somewhat concerned  (2) 

- Neutral  (3) 

- Somewhat not concerned  (4) 

- Not concerned  (5) 

Q12 On average, from April through November, how many hours a day are the horses that you own or care for on pasture?

- <1 hr  (1) 

- 1-4 hrs  (2) 

- 5-8 hrs  (3) 

- 9-12 hrs  (4) 

- 13-16 hrs  (5) 

- 16+ hrs  (6) 

| Page Break |  |
| --- | --- |

Q13 Are any of your horses or those that you care for turned out near or in **forested habitat** or **forest edge** similar to these photos during the months of April-November?

  

- Yes  (1) 

- No  (2) 

- I don't know  (3) 

| Page Break |  |
| --- | --- |

Q14 Prior to this study, how often did you conduct full body checks for ticks on horses you own, ride, or care for from April-November?

- Daily  (1) 

- Weekly  (2) 

- Monthly  (3) 

- When I remember or find a tick  (4) 

- Never  (5) 

Q15 Prior to this study, how often did you conduct partial body checks for ticks on horses you own, ride, or care for from April-November?

- Daily  (1) 

- Weekly  (2) 

- Monthly  (3) 

- When I remember or find a tick  (4) 

- Never  (5) 

Q16 What do you find challenging about conducting tick checks? (Check all that apply)

- Finding ticks in the coat  (1) 

- Knowing where to look  (2) 

- The time it takes to complete  (3) 

- Remembering to do them  (4) 

- I've never conducted tick checks before  (5) 

Q17 Which season(s) do you typically check horses(s) you own, care for, or ride for ticks? (Select all that apply)

- Spring  (1) 

- Summer  (2) 

- Fall  (3) 

- Winter  (4) 

- I don't check for ticks  (5) 

| Page Break |  |
| --- | --- |

Q18 For multiple horses in the same pasture, have you observed some horses getting more ticks, or ticks more frequently, than other horses where they are pastured?

- Yes  (1) 

- No  (2) 

- Sometimes  (3) 

- I only have one horse  (4) 

Q19 Think about the horse you own, ride, or care for that gets the most ticks. On average, how many times per year do you find ticks on this horse?

- 1-2  (1) 

- 3-5  (2) 

- 6-10  (3) 

- 11+  (4) 

- I don't know  (5) 

Q20 Think about the horse you own, ride, or care for that gets the most ticks. When you do find ticks on this horse, how many ticks do you estimate you find on each occasion?

- 1-2  (1) 

- 3-5  (2) 

- 6-10  (3) 

- 11+  (4) 

- I don't know  (5) 

| Page Break |  |
| --- | --- |

Q21 Click on the **one** area of the horse that best represents where you find the **most** ticks on horses(s) you own, ride, or care for. 

| Page Break |  |
| --- | --- |

Q22 What methods of tick control do you use on your horse or horses in your care? (Check all that apply)

- Pasture or landscape management (i.e., trimming vegetation, moving fence lines away from wooded areas, mowing, etc.)  (1) 

- Landscape chemicals (i.e., spraying chemicals in areas ticks might be)  (2) 

- On-animal control (i.e., repellents)  (3) 

- Other  (4) 

- I do not use tick control methods on horses(s) I own, ride, or care for  (5) 

Q23 What is the primary method of tick control do you use on your horse or horses in your care? 

- Pasture or landscape management (i.e., trimming vegetation, moving fence lines away from wooded areas, mowing, etc.)  (1) 

- Landscape chemicals (i.e., spraying chemicals in areas ticks might be)  (2) 

- On-animal control (i.e., repellents)  (3) 

- Other  (4) 

- I do not use tick control methods on horses(s) I own, ride, or care for  (5) 

Q24 Do you use any of the following tick control methods or products to **specifically** control or prevent ticks from biting your horses (not for fly control)? (Select all that apply)

- Equispot  (1) 

- Spray (synthetic) repellents (i.e., permethrin)  (2) 

- Spray (natural) repellents (i.e., Outsmart, Ecovet, equiderma, etc.)  (3) 

- Fipronil  (4) 

- Homemade repellent  (5) 

- I don't use any products to specifically prevent tick bites  (6) 

End of Block: Repeated Questions from Other Project

Start of Block: Horse 1

Q7 Please fill in the following information about Horse #1.

- Name  (1) __________________________________________________

- Height (hands)  (2) __________________________________________________

- Breed  (3) __________________________________________________

- Sex  (4) __________________________________________________

- Age  (5) __________________________________________________

- PA County horse resides in for the majority of the year  (6) __________________________________________________

Q8 What size fly mask does Horse #1 wear? 

- Medium (cob)  (1) 

- Large (full)  (2) 

- Extra large  (3) 

Q9 What size Shoofly Leggings does Horse #1 wear? Length is listed here, but for more detailed sizing information including top and bottom circumference AND a fitting guide video, please visit the manufacturer's website: http://shooflyleggins.com/product_info

Please note that there will NOT be any time to swap out sizes after the products arrive, so please measure your horse for an accurate size.

- Small (Length 13.5 in.)  (1) 

- Medium (Length 14.5 in.)  (2) 

- Large (Length 15 in.)  (3) 

End of Block: Horse 1

Start of Block: Horse 2

Q31 Please fill in the following information about Horse #2.

- Name  (1) __________________________________________________

- Height (hands)  (2) __________________________________________________

- Breed  (3) __________________________________________________

- Sex  (4) __________________________________________________

- Age  (5) __________________________________________________

- PA County horse resides in for the majority of the year  (6) __________________________________________________

Q32 What size fly mask does Horse #2 wear? 

- Medium (cob)  (1) 

- Large (full)  (2) 

- Extra large  (3) 

Q33 What size Shoofly Leggings does Horse #2 wear? Length is listed here, but for more detailed sizing information including top and bottom circumference AND a fitting guide video, please visit the manufacturer's website: http://shooflyleggins.com/product_info

Please note that there will NOT be any time to swap out sizes after the products arrive, so please measure your horse for an accurate size.

- Small (Length 13.5 in.)  (1) 

- Medium (Length 14.5 in.)  (2) 

- Large (Length 15 in.)  (3) 

End of Block: Horse 2

Start of Block: Horse 3

Q34 Please fill in the following information about Horse #3.

- Name  (1) __________________________________________________

- Height (hands)  (2) __________________________________________________

- Breed  (3) __________________________________________________

- Sex  (4) __________________________________________________

- Age  (5) __________________________________________________

- PA County horse resides in for the majority of the year  (6) __________________________________________________

Q35 What size fly mask does Horse #3 wear? 

- Medium (cob)  (1) 

- Large (full)  (2) 

- Extra large  (3) 

Q36 What size Shoofly Leggings does Horse #3 wear? Length is listed here, but for more detailed sizing information including top and bottom circumference AND a fitting guide video, please visit the manufacturer's website: http://shooflyleggins.com/product_info

Please note that there will NOT be any time to swap out sizes after the products arrive, so please measure your horse for an accurate size.

- Small (Length 13.5 in.)  (1) 

- Medium (Length 14.5 in.)  (2) 

- Large (Length 15 in.)  (3) 

End of Block: Horse 3
