# Supplementary material for: Barriers to entry: A participatory science study of tick identification, monitoring, and physical barriers to reduce tick bites on horses
Source: PLoS One. 2026 Oct 7;21(10):e0357053. doi: 10.1371/journal.pone.0357053 (PMC13645029; doi:10.1371/journal.pone.0357053)
Supplement: Supplementary file 4 [file pone.0357053.s004.docx]

| **Pennsylvania County** | **Participants Year 1 (N)** | **Participants Year 2 (N)** | **Total Participants (N)** |
| --- | --- | --- | --- |
| Adams | 1 | 0 | 1 |
| Allegheny | 0 | 1 | 1 |
| Beaver | 1 | 0 | 1 |
| Bedford | 0 | 1 | 1 |
| Blair | 1 | 0 | 1 |
| Bradford | 1 | 0 | 1 |
| Bucks | 1 | 0 | 1 |
| Butler | 1 | 0 | 1 |
| Cambria | 0 | 1 | 1 |
| Centre | 4 | 1 | 5 |
| Chester | 1 | 0 | 1 |
| Clearfield | 1 | 1 | 2 |
| Clinton | 0 | 1 | 1 |
| Columbia | 0 | 1 | 1 |
| Cumberland | 0 | 1 | 1 |
| Dauphin | 0 | 1 | 1 |
| Delaware | 1 | 1 | 2 |
| Elk | 1 | 1 | 2 |
| Fayette | 1 | 0 | 1 |
| Fulton | 0 | 1 | 1 |
| Juniata | 1 | 0 | 1 |
| Lancaster | 2 | 0 | 2 |
| Lawrence | 0 | 1 | 1 |
| Lebanon | 0 | 1 | 1 |
| Lycoming | 1 | 2 | 3 |
| Mercer | 1 | 0 | 1 |
| Montgomery | 1 | 0 | 1 |
| Perry | 1 | 0 | 1 |
| Warren | 0 | 1 | 1 |
| Washington | 5 | 1 | 6 |
| Westmoreland | 2 | 1 | 3 |
| York | 4 | 2 | 6 |
| **Grand Total** | **33** | **21** | **54** |
