# Supplementary material for: Barriers to entry: A participatory science study of tick identification, monitoring, and physical barriers to reduce tick bites on horses
Source: PLoS One. 2026 Oct 7;21(10):e0357053. doi: 10.1371/journal.pone.0357053 (PMC13645029; doi:10.1371/journal.pone.0357053)
Supplement: Supplementary file 5 [file pone.0357053.s006.docx]

March 10, 2026

Dina Aboelsoued, Ph.D.
Academic Editor
PLOS One

Dear Dr. Aboelsoued,

Thank you for the recent time and effort that you and the reviewers put into our manuscript, "Barriers to entry: A participatory science study of tick identification, monitoring, and physical barriers to reduce tick bites on horses,” manuscript #PONE-D-25-58073. The authors sincerely appreciate the time and comments provided by you and the reviewers. We have completed revisions and suggestions in the final version of the paper and attached both a track changes and clean version for further review. Please find responses to reviewer comments below.

*Editor Comments:*

**1. Please ensure that your manuscript meets PLOS ONE's style requirements, including those for file naming. The PLOS ONE style templates can be found at**

[**https://journals.plos.org/plosone/s/file?id=wjVg/PLOSOne_formatting_sample_main_body.pdf**](https://journals.plos.org/plosone/s/file?id=wjVg/PLOSOne_formatting_sample_main_body.pdf) **and**[**https://journals.plos.org/plosone/s/file?id=ba62/PLOSOne_formatting_sample_title_authors_affiliations.pdf**](https://journals.plos.org/plosone/s/file?id=ba62/PLOSOne_formatting_sample_title_authors_affiliations.pdf)

We have reviewed the manuscript and made the style adjustments.

**2. Please provide additional details regarding participant consent. In the ethics statement in the Methods and online submission information, please ensure that you have specified (1) whether consent was informed and (2) what type you obtained (for instance, written or verbal, and if verbal, how it was documented and witnessed). If your study included minors, state whether you obtained consent from parents or guardians. If the need for consent was waived by the ethics committee, please include this information.**

**If you are reporting a retrospective study of medical records or archived samples, please ensure that you have discussed whether all data were fully anonymized before you accessed them and/or whether the IRB or ethics committee waived the requirement for informed consent. If patients provided informed written consent to have data from their medical records used in research, please include this information.**

Thank you for this comment. This study was reviewed by the Institutional Review Board under protocol STUDY00016457 and was determined to be **not human subjects research**, and therefore did not require IRB review, informed consent, or documentation of consent. As a result, no written or verbal consent procedures were implemented.

We have clarified this in the Methods section to explicitly state the IRB determination and exemption status.

**3. PLOS requires an ORCID iD for the corresponding author in Editorial Manager on papers submitted after December 6th, 2016. Please ensure that you have an ORCID iD and that it is validated in Editorial Manager. To do this, go to ‘Update my Information’ (in the upper left-hand corner of the main menu), and click on the Fetch/Validate link next to the ORCID field. This will take you to the ORCID site and allow you to create a new iD or authenticate a pre-existing iD in Editorial Manager.**

I get this error

My ID Is

**4. Thank you for stating the following financial disclosure:
This study was funded by the College of Agriculture at Penn State Science to Practice project funding, Penn State Extension Animal Systems Unit, and supported by the USDA National Institute of Food and Agriculture and Hatch Appropriations under Project PEN04762 and Accession number 1025757.**

**Please state what role the funders took in the study. If the funders had no role, please state: "The funders had no role in study design, data collection and analysis, decision to publish, or preparation of the manuscript."
If this statement is not correct you must amend it as needed.
Please include this amended Role of Funder statement in your cover letter; we will change the online submission form on your behalf.**

Added to statement below.

**5. Thank you for stating the following in the Acknowledgments Section of your manuscript:**

**The authors thank Emily Struckhoff for tick identification assistance and the United States Department of Agriculture's National Veterinary Service Laboratory (Ames, IA) for identifying longhorned tick specimens. This study was funded by the College of Agriculture at Penn State Science to Practice project funding, Penn State Extension Animal Systems Unit, and supported by the USDA National Institute of Food and Agriculture and Hatch Appropriations under Projec PEN04762 and Accession number 1025757. Mention of trade names or commercial products in this publication is solely for the purpose of providing specific information and does not imply recommendation or endorsement by the USDA. The USDA is an equal opportunity provider and employer.**

**We note that you have provided funding information that is not currently declared in your Funding Statement. However, funding information should not appear in the Acknowledgments section or other areas of your manuscript. We will only publish funding information present in the Funding Statement section of the online submission form.**

**Please remove any funding-related text from the manuscript and let us know how you would like to update your Funding Statement. Currently, your Funding Statement reads as follows:**

**This study was funded by the College of Agriculture at Penn State Science to Practice project funding, Penn State Extension Animal Systems Unit, and supported by the USDA National Institute of Food and Agriculture and Hatch Appropriations under Project PEN04762 and Accession number 1025757.

Please include your amended statements within your cover letter; we will change the online submission form on your behalf.**

**Funding Statement**

This study was funded by the College of Agriculture at Penn State Science to Practice project funding, Penn State Extension Animal Systems Unit, and supported by the USDA National Institute of Food and Agriculture and Hatch Appropriations under Project PEN04762 and Accession number 1025757. The funders had no role in study design, data collection and analysis, decision to publish, or preparation of the manuscript.

**6. In the online submission form, you indicated that your data will be submitted to a repository upon acceptance. We strongly recommend all authors deposit their data before acceptance, as the process can be lengthy and hold up publication timelines. Please note that, though access restrictions are acceptable now, your entire minimal dataset will need to be made freely accessible if your manuscript is accepted for publication. This policy applies to all data except where public deposition would breach compliance with the protocol approved by your research ethics board. If you are unable to adhere to our open data policy, please kindly revise your statement to explain your reasoning and we will seek the editor's input on an exemption.** The datafiles are currently being processed with ScholarSphere.

**7. If the reviewer comments include a recommendation to cite specific previously published works, please review and evaluate these publications to determine whether they are relevant and should be cited. There is no requirement to cite these works unless the editor has indicated otherwise.**

This was not applicable.

***Reviewer Comments:***

***Reviewer #1***

**Lines 51-53: "In central New Jersey, seroprevalence increased from 12% among mares and 35% among yearlings in 1983 to 60% among mares and yearlings by 1985 on the same farm." -- From this quote alone, the evidence doesn't sound very compelling since it sounds like only one farm was sampled (to a general audience, at least, a farm doesn't sound very big). I looked up the study and it sounds like quite an extensive farm (which actually consists of >1 farm site). Could you add some more info to this sentence, or an additional sentence, explaining a bit more about the study (e.g., how many horses were sampled)?**

We appreciate this suggestion and have revised the sentence to provide additional context regarding the scale of the study. It now reads:

“Longitudinal studies document this expansion through rising seroprevalence rates in affected regions. On a large Standardbred breeding operation in central New Jersey comprising two farm sites and over 170 horses sampled, seroprevalence increased from 12% among mares and 35% among yearlings in 1983 to 60% among mares and yearlings by 1985–1986.”

**Lines 70-71: "Permethrin-treated clothing has shown 73.6-100% protection against various tick species in human populations" -- Are other insecticides used for tick control? How do they compare with permethrin?**

Other insecticides are used for tick control; however, these are typically applied as topical repellents rather than fabric treatments. Their modes of action differ from permethrin, and they are designed to be transient (i.e., wash off), making them not directly comparable to permethrin-treated clothing.

**Line 99: "Participants were required to be located in Pennsylvania and age 18 or older." -- Why were these the only requirements for the Year 1 portion of the study, where participants searched for ticks on their horses, while one of the requirements of the Year 2 study was "ownership of at least one horse standing ≥15.0 hands in height" (Line 132).**

In Year 1, inclusion criteria were intentionally broad to support baseline surveillance and participant training. Participants were required to be ≥18 years of age, located in Pennsylvania, complete a standardized tick-check training, and agree to perform twice-weekly tick checks and submit collected ticks. There were no requirements related to horse size, prior tick exposure, or restrictions on tick control practices, as the primary goal was to characterize tick presence and build participant confidence in surveillance.

In Year 2, inclusion criteria were more restrictive to support evaluation of a controlled intervention. Participants were required to be ≥18 years of age, own at least one horse ≥15.0 hands (to standardize barrier fit), and have documented tick exposure within the previous 12 months. Additional exclusion criteria were implemented, including refraining from the use of other tick or fly control products during the study period to avoid confounding effects. Participants were also required to comply with intervention protocols (use of fly boots and masks) in addition to continued tick surveillance and submission.

Together, these differences reflect a transition from a broad, observational design in Year 1 to a more controlled, intervention-based study in Year 2, with stricter criteria to ensure consistency and interpretability of treatment effects.

To clarify in the text, we added this “In contrast to Year 1, which used broad inclusion criteria for surveillance and training, Year 2 implemented more restrictive inclusion and exclusion criteria to support evaluation of a controlled intervention. These included requirements for prior tick exposure, standardized horse size, and restrictions on concurrent tick control product use to minimize confounding effects.”

**Lines 171-173: "Participants were instructed to conduct full-body tick checks twice weekly from April 18 to June 5, 2022, for Year 1, and from April 3 to April 30, 2023, in Year 2, with at least 48 hours between checks." -- Why were these specific months/dates chosen? Why did the time periods differ between the Year 1 and Year 2 surveys?**

We added clarification that Year 2 sampling was restricted to align with *Ixodes scapularis* phenology. This species is most active in early spring, and the shortened timeframe allowed targeted evaluation of protection during peak risk.

**Line 193: "Responses with duplicate names were filtered to keep the more complete response." -- Not sure what this means.**

This has been revised to:
“Duplicate entries were identified by name, and when individuals submitted multiple responses, the most complete submission was retained.”

**Line 200: "in species abundance" -- This should read as "in tick species abundance."**

Changed as requested.

**Line 269: "Most respondents (84.8%; n=46) turn their horses out near or in forested habitat or forest edges. " -- This is the first time forests or forest edges are mentioned in the manuscript. I would suggest introducing the concept of forests/forest edges and tick habitats, and their relationship to horses, in the introduction. It would better explain why these habitats were asked about in your survey.**

This was added to the end of the first paragraph in the introduction “Equine tick exposure can be influenced by landscape features. Many tick species prefer forested and edge habitats, so tick bite risk can increase near forested areas, woodland edges, and in settings where horses are ridden or turned out in wooded or transitional habitats, where tick densities tend to be greatest.”

**Figure 2: The colour bars need labels. I know, from the figure caption, what the colours mean, but can't see the little numbers in the colour bars very well.**

Labels have been added to the color bars.

**Lines 309-310: "Increased confidence in performing tick checks on horses was rated on the same 1-5 scale, with a mean increase of 3.81" -- this sounds like a big increase! What was the mean confidence level at the beginning of the study?**

This was worded poorly; there was only one question and it asked participants to rate their increase in confidence. “Increase” has been changed to “score” and the scale descriptions were added. The question itself read: “Please rate how much your confidence in performing tick checks on horses increased as a result of the project” followed by a 1 to 5 scale with 1 being none at all and 5 being a great deal.

**Line 312: "Most respondents (86.1%) report that they intend to continue..." -- Change verbs to past tense.**

Changed as requested.

**Lines 362-363: "Of the total ticks, 230 were returned for identification in Year 1, and 51 were returned in Year 2" -- why were not all the ticks collected not returned for identification?**

Tick return depended on participant compliance, and not all participants submitted collected specimens, which is a known limitation of community-based studies.

**Line 363: "All returned ticks were adults." -- Was this expected? Could this be a result of adults just being easier to see than nymphs?**

While it is true that adults will be easier to see than nymphs, most ticks of medical and veterinary importance feed on large animals in the adult stage. In addition, the timing of this project targeted the adult life stages, so it would have been unlikely for participants to find nymphs, though not impossible.

**Lines 553-554: "The detection of H. longicornis in 2023 collections, although numerically small, represents a significant finding..." -- This is important, and the finding of this tick is mentioned in several areas of the manuscript. What is the potential health significance of this tick to horses? Can it affect humans? Has it been found in Pennsylvania before, just not in Clinton? If so, where and when?**

The detection of *Haemaphysalis longicornis* in 2023, although limited in number, is significant given its status as an invasive and expanding tick in the United States. This species readily feeds on large mammals, including horses, where high infestations can cause irritation, blood loss, and, in severe cases, anemia, though its role in transmitting equine pathogens in the U.S. remains unclear. It is also known to bite humans and is considered a potential emerging public health concern, as populations in the U.S. have tested positive for pathogens such as *Anaplasma phagocytophilum* and *Borrelia burgdorferi*, although confirmed transmission to humans in the field has not been established domestically. While *H. longicornis* has been confirmed to not transmit *A. phagocytophilum* or *B. burgdorferi* in laboratory settings (Levin et al. 2021, Breuner et al 2019), it can transmit the causative agent of Rocky Mountain spotted fever (*Rickettsia rickettsii*). In Pennsylvania, *H. longicornis* was first detected in 2018 on a white-tailed deer and has since been reported across multiple counties, indicating that this finding likely represents continued geographic spread rather than initial introduction, and may reflect a new county-level detection within the study area.

***Reviewer #2:***

**I also suggest adding a table or two reporting the survey responses from each year. Having these only reported in the text makes it difficult to find and compare between years. As a side note, the current representation of sample size and percentages could be confusing to readers. In other articles, statements are presented along the lines of “most horses (N=XX; YY%) have …” where XX is the number of horse for which the statement is true and (XX/total sample size) x 100 = YY%.**

The authors did consider adding such a table, the two surveys did not ask the exact same questions, so the table was too complicated. The authors thought that writing it out was less confusing to readers. As for the sample size and percentages, we do note in the M&M how data is presented for surveys. “Results from all surveys are presented as a percentage of the number of respondents for each question. Since not all respondents answered each question, the number of respondents is also presented. When multiple selections were allowed, the data presented are the percentage of respondents who chose that selection.”

We have added that statement into Results as well to remind readers how the data is presented for surveys. This author group has successfully published several other papers with survey results and we have historically presented n as the number of respondents who answered the question.

**Line 99 and 131-134. Were the same study inclusion/exclusion criteria used for participation in each year? It is unclear from the text. Please clarify and explain differences.**

In Year 1, inclusion criteria were intentionally broad to support baseline surveillance and participant training. Participants were required to be ≥18 years of age, located in Pennsylvania, complete a standardized tick-check training, and agree to perform twice-weekly tick checks and submit collected ticks. There were no requirements related to horse size, prior tick exposure, or restrictions on tick control practices, as the primary goal was to characterize tick presence and build participant confidence in surveillance.

In Year 2, inclusion criteria were more restrictive to support evaluation of a controlled intervention. Participants were required to be ≥18 years of age, own at least one horse ≥15.0 hands (to standardize barrier fit), and have documented tick exposure within the previous 12 months. Additional exclusion criteria were implemented, including refraining from the use of other tick or fly control products during the study period to avoid confounding effects. Participants were also required to comply with intervention protocols (use of fly boots and masks) in addition to continued tick surveillance and submission.

Together, these differences reflect a transition from a broad, observational design in Year 1 to a more controlled, intervention-based study in Year 2, with stricter criteria to ensure consistency and interpretability of treatment effects.

To clarify in the text, we added this “In contrast to Year 1, which used broad inclusion criteria for surveillance and training, Year 2 implemented more restrictive inclusion and exclusion criteria to support evaluation of a controlled intervention. These included requirements for prior tick exposure, standardized horse size, and restrictions on concurrent tick control product use to minimize confounding effects.”

**Lines 196-204. This is not a fair comparison as Year 2 data only span a single month while Year 1 spanned a bit over 2 months. Different tick species have different phenology such that differences in collections between the years may simply be due to different timeframes, as acknowledged in the Discussion (lines 535-547). Subsetting the data from Year 1 to only collections in April would enable more accurate comparisons. As presented (Lines 373-380), comparisons of species abundance and diversity are misleading.**

We appreciate this comment and agree that differences in sampling duration and seasonal timing influence species composition due to tick phenology. Importantly, this comparison was not intended to represent a controlled, like-for-like evaluation between years, but rather to demonstrate how shifting the sampling window can result in different observed species assemblages. We have clarified this point in the manuscript and tempered language around direct comparisons. We have added the following text to the results

“Species composition differed between years (χ² = 513.9, df = 3, p < 0.001); however, these differences should be interpreted in the context of differing sampling timeframes and seasonal tick phenology rather than as a direct year-to-year comparison.”

**Lines 207-209. A paired t-test is not the correct test for this comparison because different horses were enrolled in Year 1 and 2. Using only the horses that were enrolled in both years would make the paired test appropriate, but neglects treatment status which could influence attachment location. Please re-assess comparisons and update results.**

We agree that the paired t-test was not appropriate. We re-analyzed attachment-site distributions using Fisher’s exact test with Monte Carlo simulation because several categories had small counts and zero values. The Methods and Results were updated accordingly.

**Lines 210-245. It is unclear why Year 1 data were included in these models, since no interventions were performed, and how the difference in sampling duration was accounted for. Additionally, it is unclear what data from Year 2 were included. Please clarify.**

We appreciate the reviewer for identifying this point. Inclusion of Year 1 data in the model description was a typographical error. The intervention models were conducted using Year 2 data only, as this was the period in which treatments were applied and evaluated. Year 1 data were not included in these analyses. We have corrected the text to clearly state that only Year 2 observations were used and clarified the dataset included in the models to avoid confusion.

**Line 264. Were participants excluded from participation if they did not answer any of the first three questions correctly (i.e., did not appear to understand the requirements of the study)? Could the surveys be included as supplemental material so readers can see the structure and wording of questions? This could inform the design of future surveys.**

They were not excluded from the study as we did not collect their names in the pre-survey. We were confident in the high percent that did get the questions correct that they understood the requirements. In addition, we reiterated the requirements in the data collection instruction sheets that each participant received. Surveys will be included as supplemental material.

**Lines 359-366. There are contradictions between numbers stated here in the text and those presented in Tables 102. For example, the text states that there were 51 ticks in 2022 after removing the anomalously high results from a single horse. This adjustment is only included in Table 2, but the adjusted total here is 101. The text indicates this anomalously high collection was 226 ticks while the footnotes for Table 2 indicate it was 286 ticks.**

We thank the reviewer for identifying these inconsistencies. These discrepancies resulted from errors in reporting adjusted totals and from anomalous collection values. We have carefully reviewed and corrected all affected values to ensure consistency between the text and tables. In addition, we clarified the distinction between Year 1 and Year 2 tick totals and ensured that the description of the anomalously high collection aligns with the corrected table footnotes.

**Line 385-386. Please provide the statistical test details for this statement of significance. Assuming attachment sites do not vary across species (unknown from the presented data), a chi-squared test for proportions across body locations is likely a valid test for this comparison.**

This was added to table 2, and to the statistical methods. Because several attachment-site categories had small counts or zero observations, differences in anatomical distribution between years were evaluated using Fisher’s exact test with Monte Carlo simulation. All comparisons were conducted using adjusted Year 1 counts, in which ticks collected from a single outlier horse (Z-score = 4.19) were removed, and observed counts from Year 2. To further assess differences at individual body locations, post hoc comparisons were conducted using Fisher’s exact tests for each anatomical region. P-values from these tests were adjusted for multiple comparisons using the Benjamini–Hochberg procedure.

**Lines 406-410. It is unclear if and why observations from Year 1 were included in these comparisons and analyses**

Thank you for noting this. Observations from Year 1 were not included in these comparisons or analyses. This was a typographical error in the manuscript, and the text has been revised to clarify that only Year 2 data were used for these analyses.

**Lines 460-464. Given the variability across weeks and the non-significance of this covariate in the GLMM, did the authors consider dropping this covariate from the GLMM?.**

We appreciate this suggestion. Although week was not statistically significant in the GLMM, we retained it in the model because it represents a biologically and temporally meaningful source of variation in tick exposure. Tick activity is known to vary over short time scales, and inclusion of week accounts for potential temporal confounding across the study period. Importantly, removing week from the model did not change the direction or significance of treatment effects (data not shown). Therefore, we retained week to improve model robustness and biological interpretability.

**Lines 533-534. The data presented in Table 4 does not provide evidence that untreated barriers were better (or different) than treated barriers. The authors need to statistically compare the fitted coefficients for these barrier treatments to assess significant differences. The information presented compared each physical barrier to no barrier.**

We thank the reviewer for this important comment. We agree that our original wording implied a direct comparison between treated and untreated barrier conditions that was not statistically evaluated. The model presented compares each barrier treatment to the no-treatment reference group and does not test differences between barrier types.

In response, we have revised the manuscript to remove the statement that untreated barriers provided “the best protection.” The text now reflects that both treated and untreated physical barriers reduced tick encounters relative to no treatment, but that differences between barrier types were not statistically supported. We also clarified this limitation in the Results and Discussion to ensure that interpretation aligns with the statistical framework presented.

“Physical barriers worn by the horse reduced tick encounters relative to no treatment, with both treated and untreated barriers showing evidence of protection (Table 3), although differences between barrier types were not statistically supported.”

**Table 1. Please use the same number of significant figures for presented percentages for consistency. Also, with the difference in timeframes between study years, the calculated change is misleading. There is a relatively high proportion of ticks of unknown species. Were these specimens damaged such as to prevent morphological identification? Were alternative methods considered to speciate these ticks?**

The numerical change has been made as requested. We agree that differences in sampling duration may influence interpretation of percent change values. We have retained these calculations to describe differences between sampling periods but have clarified in the table footnotes that these should be interpreted cautiously in the context of differing timeframes and phenology. In terms of unknown species, all specimens returned were identified. Unknowns largely referred to ticks that were counted by participants, but were not returned for identification. Some specimens were not able to be identified because only a portion of the tick was returned. We have added this to the footnotes ^c^ Some returned specimens were damaged (e.g., crushed or incomplete), limiting accurate identification. Additionally, not all participants submitted collected ticks, and species identification could therefore not be conducted for all observations.

**Table 2. Why are the counts of adjusted tick numbers sometimes larger than the non-adjusted counts across body locations? Counts should be less than or equal to the non-adjusted counts after removing the data for the collections from the anomalously high horse.**

We thank the reviewer for catching this error. The discrepancy was due to a data-entry error in which Year 2 values were inadvertently placed in the adjusted Year 1 column. This has been corrected, and all adjusted counts now appropriately reflect the removal of the anomalous observations and are less than or equal to the corresponding non-adjusted counts.

Again, it is with many thanks that I respond to your comments and concerns regarding this manuscript and offer my sincere appreciation for the time and effort you put forth in the editorial comments which, no doubt, will improve the document as a whole.

Sincerely,

Erika T. Machtinger, PhD

Pennsylvania State University
