# Supplementary material for: Barriers to entry: A participatory science study of tick identification, monitoring, and physical barriers to reduce tick bites on horses
Source: PLoS One. 2026 Oct 7;21(10):e0357053. doi: 10.1371/journal.pone.0357053 (PMC13645029; doi:10.1371/journal.pone.0357053)
Supplement: Supplementary file 9 [file pone.0357053.s007.docx]

March 10, 2026

Dina Aboelsoued, Ph.D.
Academic Editor
PLOS One

Dear Dr. Aboelsoued,

Thank you for the recent time and effort that you and the reviewers put into our manuscript, "Barriers to entry: A participatory science study of tick identification, monitoring, and physical barriers to reduce tick bites on horses,” manuscript #PONE-D-25-58073. The authors sincerely appreciate the time and comments provided by you and the reviewers. We have incorporated your suggestions into the final version of the paper and attached both a track changes and a clean version for further review. Please find responses to reviewer comments below.

Figure 2: The colour bars need labels. I know, from the figure caption, what the colours mean, but can't see the little numbers in the colour bars very well.

--> From what I can see on my end, Figure 2 is still not clearly labelled. I don't know if they did update the figure and I just can't see/access it? I will leave this up to the editor to make the final judgement on. **The labels were provided on Figure 2. We double checked the submission and they were there.**

Lines 553-554: "The detection of H. longicornis in 2023 collections, although numerically small, represents a significant finding..." -- This is important, and the finding of this tick is mentioned in several areas of the manuscript. What is the potential health significance of this tick to horses? Can it affect humans? Has it been found in Pennsylvania before, just not in Clinton? If so, where and when?

--> the authors answered these questions well in their direct response to the edit, but did not actually make the necessary modifications in the text itself. **We have added the following sentence to the discussion “This parthenogenetic tick feeds on large ungulates and, although it has not been associated with transmission of equine pathogens, heavy infestations have caused blood loss, anemia, reduced productivity, and mortality in cattle, highlighting the potential impact of high tick burdens on large animal hosts [33].” As well as specifying that this tick had been previously found in Pennsylvania, this was just a new detection in Clinton County.**

I am still a bit concerned about the calculation of change in tick species abundance between the years given the difference in sampling periods. Thank you to the authors for adding footnotes and discussion highlighting that differences are due to phenological differences. I suggest adding this note to the Methods to provide consistency and clarity for the readers and reduce overstating the significance of differences. Differences in abundance could also be related to different locations between the years (habitats, ecology, etc) and recommend adding this to the appropriate sections. **This has been added to the Tick Species section of the methods “Because sampling periods differed between years, percent changes were used only as directional comparisons relative to the Year 1 baseline and should not be interpreted as standardized estimates of interannual change. Differences in species abundance may reflect seasonal tick phenology associated with the shorter, earlier sampling period in Year 2, as well as differences in sampling locations, habitat characteristics, and local ecological conditions between years.” And to the discussion we added “years reflects not only location and microecology differences, but likely primarily…”**

I also suggest, if possible, to add a figure (e.g., barplot) illustrating the percent responses from each year’s survey when questions were the same. Since the results are presented in separate sections for each survey, it is a bit difficult to compare presented percentages. A figure would provide a side-by-side visualization that readers can refer to, especially for the comparisons presented in the Discussion. **Only nine questions were identical across the two surveys. After reviewing these items, we determined that they did not provide a sufficiently comprehensive or informative basis for a meaningful comparative figure. We were concerned that presenting these limited items visually could overemphasize comparisons that were not central to the study’s objectives. Therefore, we elected not to add the proposed figure and instead retained the year-specific presentation of the results.**

Line 39-43. The statements that tick attachment site remained consistent across body regions contradicts the findings in the Results (Lines 416-417) that tick attachment site varied significantly across years. Please reconcile or reword to clarify. **This change has been reworded to “Tick attachment occurred across eight body locations and differed significantly between years. ” in the abstract.**

Line 50-51. Anaplasma phagocytophilum is the causative agent of anaplasmosis in humans too, not just equids. I suggest rewording to reduce potential confusion. **We eliminated “equine” in front of anaplasmosis to clarify.**

Line 62. I suggest adding sample sizes to contextualize these percentages. These are high values (66-100%), but sample sizes were relatively small (10-50). **Thank you for this suggestion. We have retained the original wording because the cited percentages are used only to illustrate geographic variation in reported seroprevalence, rather than to compare study precision or derive a pooled estimate.**

Lines 92-94. Please re-word this statement. Only data from Year 2 was used to assess control measure efficacy while this statement makes it appear that Year 1 data were also included. As indicated in section headings, Year 1 was more aimed at developing confidence while Year 2 was more for assessing control. **We moodified for clarity to “During Year 1, participants conducted systematic tick monitoring to develop proficiency with study protocols and characterize tick presence and attachment patterns on horses. During Year 2, participants implemented physical barrier interventions while continuing standardized tick surveillance, and only Year 2 data were used to evaluate intervention efficacy. Together, the two study phases generated information on tick species affecting horses in Pennsylvania, assessed the effectiveness of physical barriers for equine tick prevention, and demonstrated the feasibility of community-based research in equine health.”**

Lines 104-107, 134-142. How long and for what period of time were participants recruited for each study year? Readers may be curious about recruitment details. **We added the months that recruitment took place in the Materials and Methods section.**

Line 223. Could you provide more details on this outlier horse? Was it in a particular habitat that was different than other horses? Was it the only horse sampled in a county? Was it one of the 8 that was in both years that you could compare counts across time? Were all the ticks submitted at one collection or were they collected over multiple checks? **We reviewed the characteristics of this horse and considered whether additional description would improve interpretation of the results. Because this represented a single observation, we did not identify evidence that it reflected a unique habitat, county, sampling history, or other systematic factor relevant to the study objectives. Including these details would place undue emphasis on an isolated observation without changing the interpretation of the findings. Our intent was simply to acknowledge the presence of an unusually high tick count while focusing the analysis on population-level patterns. Therefore, we have elected not to expand this section further.**

Lines 280-282. Since this information is presented in the Methods, it is not necessary to repeat it here. **These sentences have been deleted as requested.**

Line 380. How did the tick abundance and diversity compare between years for the 8 horses that participated in both study years? This could supplement the discussion and interpretation of differences between years for the full dataset. **Thank you for this suggestion. We agree that repeated observations on the same horses could provide useful longitudinal insight in a larger study. However, only eight horses participated in both study years, representing a small, non-random subset of the study population. Given the limited sample size and the differences in environmental conditions and tick phenology between years, we do not believe that comparisons within these individuals would provide robust or generalizable conclusions. Because such analyses would be exploratory and potentially overinterpreted, we elected to focus on the population-level comparisons that align with the study objectives and design.**

Line 381-382. Please check your numbers. Removing 286 ticks from Year 1’s total (332) results in 46 adjusted ticks, not 51.**This adjustment has been made.**

Line 451-452. An odds ratio of 0.49 translates to a 51% reduction in tick encounters. Please correct. **This adjustment has been made.**

Line 484-485. Please revisit the description of “effect uncertain”. There appear to be three conditions that could fulfil the definition (ARR ≥15% OR NNT ≤7; 95% CI for OR includes 1.0; ARR includes 0) with the first and third contradictory. **Yes, this was a copy and paste error. This has been revised to be “Effect uncertain: the estimated effect did not meet the prespecified threshold for clinical importance (ARR <15% and NNT >7), or the confidence intervals indicated statistical imprecision by including 1.0 for the OR or 0 for the ARR.”**

Line 510-511. Please mention the difference in sampling duration between these years. Otherwise, incorrect conclusions can be drawn about tick population dynamics and risk in PA, especially for readers who only skim through a single section. **The sampling period was added “Sampling was conducted over seven weeks from April 18 to June 5 in 2022 and four weeks from April 3 to April 30 in 2023.”**

Lines 521-523. Another factor is the difference in sample sizes between the studies. The cited survey had >500 responses while the surveys in Year 1 and 2 each had an order of magnitude fewer responses (40-50). **Revised this section to say “The reason for the lower level of concern reported in Year 1 is unclear but may reflect differences in participants’ prior experience with tick-borne disease or the substantially smaller sample sizes in the current study, with approximately 40–50 respondents in each year.”**

Line 554. Please explain the statement of “lower-than-expected tick counts”. To which year is being referred? How does this relate to the frequent tick checks (daily or 2-6 times/week) mentioned in the previous lines? **This was referring to participant perceptions. This has been clarified to “Participants may have expected to collect more ticks than they ultimately found, and the lower-than-expected tick recovery may have contributed to this behavioral change by creating a false sense of security regarding the likelihood of tick bites.”**

Line 617. Since you did not statistically compare treated vs. untreated barriers, it is not appropriate to state one had “superior performance” compared to the other. Commenting on the certainty (i.e., 95% CI) of fitted estimates for each would be consistent with the presented analyses. **Thank you for this comment. We agree that our original wording implied a direct statistical comparison between untreated and permethrin-treated barriers that was not conducted. We have revised the statement to describe the fitted estimates and it now reads “The fitted estimates indicated that both untreated and permethrin-treated barriers reduced blacklegged tick attachment. These findings suggest that physical exclusion alone may contribute to protection against blacklegged ticks, as has been reported for stable flies.[”**

Lines 638-639. While the presented seasons align with known phenology of blacklegged tick lifestages, not all seasons were included in the study. Therefore, I suggest not presenting these as conclusions from the study itself. **The language of this section was modified to not be as specific with seasonality and instead include “seasonal risk.”**

Table 5. I suggest not using “prevalence” in the last column heading. This could be confused for infection prevalence instead of the percent of hoses with ticks that week. This column is also redundant with column three (“observations with tick presence”) and is not needed. Also, since presence and absence are mutually exclusive, please consider the value of presenting both in this table. Consider adding a column indicating the number of horses with observation each week. **We eliminated the weekly tick prevalence column and added the N of horses observed each week, as well as an explanatory footnote.**

Again, it is with many thanks that I respond to your comments and concerns regarding this manuscript and offer my sincere appreciation for the time and effort you put forth in the editorial comments which, no doubt, will improve the document as a whole.

Sincerely,

Erika T. Machtinger, PhD

Pennsylvania State University
