# Supplementary material for: Barriers to entry: A participatory science study of tick identification, monitoring, and physical barriers to reduce tick bites on horses
Source: PLoS One. 2026 Oct 7;21(10):e0357053. doi: 10.1371/journal.pone.0357053 (PMC13645029; doi:10.1371/journal.pone.0357053)
Supplement: Peer review history

# Peer review: Decision Letter 0

**DOI:** 10.1371/journal.pone.0357053.r001

**Authors:** Dina Aboelsoued

23 Mar 2026

Dear Dr. Machtinger,

Your comprehensive investigation on "Barriers to entry: A participatory science study of tick identification, monitoring, and physical barriers to reduce tick bites on horses" has a somewhat clear presentation of information, But it needs some corrections to achieve the best presentation. Kindly find the reviewer comments and provide your response.

Please submit your revised manuscript by May 07 2026 11:59PM. If you will need more time than this to complete your revisions, please reply to this message or contact the journal office at plosone@plos.org. When you're ready to submit your revision, log on to https://www.editorialmanager.com/pone/ and select the 'Submissions Needing Revision' folder to locate your manuscript file.

- A letter that responds to each point raised by the academic editor and reviewer(s). You should upload this letter as a separate file labeled 'Response to Reviewers'.
- A marked-up copy of your manuscript that highlights changes made to the original version. You should upload this as a separate file labeled 'Revised Manuscript with Track Changes'.
- An unmarked version of your revised paper without tracked changes. You should upload this as a separate file labeled 'Manuscript'.

If you would like to make changes to your financial disclosure, please include your updated statement in your cover letter. Guidelines for resubmitting your figure files are available below the reviewer comments at the end of this letter.

If applicable, we recommend that you deposit your laboratory protocols in protocols.io to enhance the reproducibility of your results. Protocols.io assigns your protocol its own identifier (DOI) so that it can be cited independently in the future. For instructions see: https://journals.plos.org/plosone/s/submission-guidelines#loc-laboratory-protocols. Additionally, PLOS ONE offers an option for publishing peer-reviewed Lab Protocol articles, which describe protocols hosted on protocols.io. Read more information on sharing protocols at https://plos.org/protocols?utm_medium=editorial-email&utm_source=authorletters&utm_campaign=protocols.

We look forward to receiving your revised manuscript.

Kind regards,

Dina Aboelsoued, Ph.D.

Academic Editor

PLOS One

Journal Requirements:

When submitting your revision, we need you to address these additional requirements.

1. Please ensure that your manuscript meets PLOS ONE's style requirements, including those for file naming. The PLOS ONE style templates can be found at

https://journals.plos.org/plosone/s/file?id=wjVg/PLOSOne_formatting_sample_main_body.pdf and

https://journals.plos.org/plosone/s/file?id=ba62/PLOSOne_formatting_sample_title_authors_affiliations.pdf

2. Please provide additional details regarding participant consent. In the ethics statement in the Methods and online submission information, please ensure that you have specified (1) whether consent was informed and (2) what type you obtained (for instance, written or verbal, and if verbal, how it was documented and witnessed). If your study included minors, state whether you obtained consent from parents or guardians. If the need for consent was waived by the ethics committee, please include this information.

If you are reporting a retrospective study of medical records or archived samples, please ensure that you have discussed whether all data were fully anonymized before you accessed them and/or whether the IRB or ethics committee waived the requirement for informed consent. If patients provided informed written consent to have data from their medical records used in research, please include this information.

3. PLOS requires an ORCID iD for the corresponding author in Editorial Manager on papers submitted after December 6th, 2016. Please ensure that you have an ORCID iD and that it is validated in Editorial Manager. To do this, go to ‘Update my Information’ (in the upper left-hand corner of the main menu), and click on the Fetch/Validate link next to the ORCID field. This will take you to the ORCID site and allow you to create a new iD or authenticate a pre-existing iD in Editorial Manager.

4. Thank you for stating the following financial disclosure:

This study was funded by the College of Agriculture at Penn State Science to Practice project funding, Penn State Extension Animal Systems Unit, and supported by the USDA National Institute of Food and Agriculture and Hatch Appropriations under Project PEN04762 and Accession number 1025757.

Please state what role the funders took in the study. If the funders had no role, please state: "The funders had no role in study design, data collection and analysis, decision to publish, or preparation of the manuscript."

If this statement is not correct you must amend it as needed.

Please include this amended Role of Funder statement in your cover letter; we will change the online submission form on your behalf.

5. Thank you for stating the following in the Acknowledgments Section of your manuscript:

The authors thank Emily Struckhoff for tick identification assistance and the United States Department of Agriculture's National Veterinary Service Laboratory (Ames, IA) for identifying longhorned tick specimens. This study was funded by the College of Agriculture at Penn State Science to Practice project funding, Penn State Extension Animal Systems Unit, and supported by the USDA National Institute of Food and Agriculture and Hatch Appropriations under Projec PEN04762 and Accession number 1025757. Mention of trade names or commercial products in this publication is solely for the purpose of providing specific information and does not imply recommendation or endorsement by the USDA. The USDA is an equal opportunity provider and employer.

We note that you have provided funding information that is not currently declared in your Funding Statement. However, funding information should not appear in the Acknowledgments section or other areas of your manuscript. We will only publish funding information present in the Funding Statement section of the online submission form.

Please remove any funding-related text from the manuscript and let us know how you would like to update your Funding Statement. Currently, your Funding Statement reads as follows:

This study was funded by the College of Agriculture at Penn State Science to Practice project funding, Penn State Extension Animal Systems Unit, and supported by the USDA National Institute of Food and Agriculture and Hatch Appropriations under Project PEN04762 and Accession number 1025757.

Please include your amended statements within your cover letter; we will change the online submission form on your behalf.

6. In the online submission form, you indicated that your data will be submitted to a repository upon acceptance. We strongly recommend all authors deposit their data before acceptance, as the process can be lengthy and hold up publication timelines. Please note that, though access restrictions are acceptable now, your entire minimal dataset will need to be made freely accessible if your manuscript is accepted for publication. This policy applies to all data except where public deposition would breach compliance with the protocol approved by your research ethics board. If you are unable to adhere to our open data policy, please kindly revise your statement to explain your reasoning and we will seek the editor's input on an exemption.

7. If the reviewer comments include a recommendation to cite specific previously published works, please review and evaluate these publications to determine whether they are relevant and should be cited. There is no requirement to cite these works unless the editor has indicated otherwise.

[Note: HTML markup is below. Please do not edit.]

Reviewers' comments:

Reviewer's Responses to Questions

**Comments to the Author**

1. Is the manuscript technically sound, and do the data support the conclusions?

Reviewer #1: Yes

Reviewer #2: Partly

**********

2. Has the statistical analysis been performed appropriately and rigorously?

Reviewer #1: Yes

Reviewer #2: No

**********

3. Have the authors made all data underlying the findings in their manuscript fully available?

The PLOS Data policy

Reviewer #1: Yes

Reviewer #2: No

**********

4. Is the manuscript presented in an intelligible fashion and written in standard English?

Reviewer #1: Yes

Reviewer #2: Yes

**********

Reviewer #1: I found this study to be well explained and thorough. The findings are interesting, especially the discovery of H. longicornis in a new area of Pennsylvania. I suggest only minor revisions.

Lines 51-53: "In central New Jersey, seroprevalence increased from 12% among mares and 35% among yearlings in 1983 to 60% among mares and yearlings by 1985 on the same farm." -- From this quote alone, the evidence doesn't sound very compelling since it sounds like only one farm was sampled (to a general audience, at least, a farm doesn't sound very big). I looked up the study and it sounds like quite an extensive farm (which actually consists of >1 farm site). Could you add some more info to this sentence, or an additional sentence, explaining a bit more about the study (e.g., how many horses were sampled)?

Lines 70-71: "Permethrin-treated clothing has shown 73.6-100% protection against various tick species in human populations" -- Are other insecticides used for tick control? How do they compare with permethrin?

Line 99: "Participants were required to be located in Pennsylvania and age 18 or older." -- Why were these the only requirements for the Year 1 portion of the study, where participants searched for ticks on their horses, while one of the requirements of the Year 2 study was "ownership of at least one horse standing ≥15.0 hands in height" (Line 132).

Lines 171-173: "Participants were instructed to conduct full-body tick checks twice weekly from April 18 to June 5, 2022, for Year 1, and from April 3 to April 30, 2023, in Year 2, with at least 48 hours between checks." -- Why were these specific months/dates chosen? Why did the time periods differ between the Year 1 and Year 2 surveys?

Line 193: "Responses with duplicate names were filtered to keep the more complete response." -- Not sure what this means.

Line 200: "in species abundance" -- This should read as "in tick species abundance."

Line 269: "Most respondents (84.8%; n=46) turn their horses out near or in forested habitat or forest edges. " -- This is the first time forests or forest edges are mentioned in the manuscript. I would suggest introducing the concept of forests/forest edges and tick habitats, and their relationship to horses, in the introduction. It would better explain why these habitats were asked about in your survey.

Figure 2: The colour bars need labels. I know, from the figure caption, what the colours mean, but can't see the little numbers in the colour bars very well.

Lines 309-310: "Increased confidence in performing tick checks on horses was rated on the same 1-5 scale, with a mean increase of 3.81" -- this sounds like a big increase! What was the mean confidence level at the beginning of the study?

Line 312: "Most respondents (86.1%) report that they intend to continue..." -- Change verbs to past tense.

Lines 362-363: "Of the total ticks, 230 were returned for identification in Year 1, and 51 were returned in Year 2" -- why were not all the ticks collected not returned for identification?

Line 363: "All returned ticks were adults." -- Was this expected? Could this be a result of adults just being easier to see than nymphs?

Lines 553-554: "The detection of H. longicornis in 2023 collections, although numerically small, represents a significant finding..." -- This is important, and the finding of this tick is mentioned in several areas of the manuscript. What is the potential health significance of this tick to horses? Can it affect humans? Has it been found in Pennsylvania before, just not in Clinton? If so, where and when?

Reviewer #2: Machtinger et al. provide the results of surveys to horse owners in Pennsylvania, given in two different years (2022, 2023), aimed at tick surveillance and control practices. In 2023, the authors distributed physical barriers (permethrin-treated or untreated fly boots and fly mask) to some participants to compare their efficacy for reducing tick presence. They found variation in the species collected across years, but consistency in attachment sites and evidence that physical barriers reduced tick attachments. These, along with survey response summaries, are important findings for understanding tick control for equines. However, I have some concerns about the statistical analyses. Comparisons were made across timeframes without acknowledging different sampling periods, leading to biased comparisons. Numbers reported in tables and texts differed. It was also unclear if data from both years were included to assess the efficacy of the physical barriers or just the second year when physical barriers were distributed.

I also suggest adding a table or two reporting the survey responses from each year. Having these only reported in the text makes it difficult to find and compare between years. As a side note, the current representation of sample size and percentages could be confusing to readers. In other articles, statements are presented along the lines of “most horses (N=XX; YY%) have …” where XX is the number of horse for which the statement is true and (XX/total sample size) x 100 = YY%.

The authors indicated that all relevant data will be made available upon acceptance, but it is not currently available as I performed my review.

I have included some additional point-by-point comments and suggestions to improve the clarity and rigor of this manuscript.

Line 99 and 131-134. Were the same study inclusion/exclusion criteria used for participation in each year? It is unclear from the text. Please clarify and explain differences.

Lines 196-204. This is not a fair comparison as Year 2 data only span a single month while Year 1 spanned a bit over 2 months. Different tick species have different phenology such that differences in collections between the years may simply be due to different timeframes, as acknowledged in the Discussion (lines 535-547). Subsetting the data from Year 1 to only collections in April would enable more accurate comparisons. As presented (Lines 373-380), comparisons of species abundance and diversity are misleading.

Lines 207-209. A paired t-test is not the correct test for this comparison because different horses were enrolled in Year 1 and 2. Using only the horses that were enrolled in both years would make the paired test appropriate, but neglects treatment status which could influence attachment location. Please re-assess comparisons and update results.

Lines 210-245. It is unclear why Year 1 data were included in these models since no interventions were performed and how the difference in sampling duration was accounted for. Additionally, it is unclear what data from Year 2 were included. Please clarify.

Line 264. Were participants excluded from participation if they did not answer any of the first three questions correctly (i.e., did not appear to understand the requirements of the study)? Could the surveys be included as supplemental material so readers can see the structure and wording of questions? This could inform the design of future surveys.

Lines 359-366. There are contradictions between numbers stated here in the text and those presented in Tables 102. For example, the text states that there were 51 ticks in 2022 after removing the anomalously high results from a single horse. This adjustment is only included in Table 2, but the adjusted total here is 101. The text indicates this anomalously high collection was 226 ticks while the footnotes for Table 2 indicate it was 286 ticks.

Line 385-386. Please provide the statistical test details for this statement of significance. Assuming attachment sites do not vary across species (unknown from the presented data), a chi-squared test for proportions across body locations is likely a valid test for this comparison.

Lines 406-410. It is unclear if and why observations from Year 1 were included in these comparisons and analyses.

Lines 460-464. Given the variability across weeks and the non-significance of this covariate in the GLMM, did the authors consider dropping this covariate from the GLMM?

Lines 533-534. The data presented in Table 4 does not provide evidence that untreated barriers were better (or different) than treated barriers. The authors need to statistically compare the fitted coefficients for these barrier treatments to assess significant differences. The information presented compared each physical barrier to no barrier.

Table 1. Please use the same number of significant figures for presented percentages for consistency. Also, with the difference in timeframes between study years, the calculated change is misleading. There is a relatively high proportion of ticks of unknown species. Were these specimens damaged such as to prevent morphological identification? Were alternative methods considered to speciate these ticks?

Table 2. Why are the counts of adjusted tick numbers sometimes larger than the non-adjusted counts across body locations? Counts should be less than or equal to the non-adjusted counts after removing the data for the collections from the anomalously high horse.

**********

what does this mean?). If published, this will include your full peer review and any attached files.

If you choose “no”, your identity will remain anonymous but your review may still be made public.

**Do you want your identity to be public for this peer review?**  For information about this choice, including consent withdrawal, please see our Privacy Policy

Reviewer #1: No

Reviewer #2: No

**********

[NOTE: If reviewer comments were submitted as an attachment file, they will be attached to this email and accessible via the submission site. Please log into your account, locate the manuscript record, and check for the action link "View Attachments". If this link does not appear, there are no attachment files.]

To ensure your figures meet our technical requirements, please review our figure guidelines: https://journals.plos.org/plosone/s/figures

You may also use PLOS’s free figure tool, NAAS, to help you prepare publication quality figures: https://journals.plos.org/plosone/s/figures#loc-tools-for-figure-preparation.

NAAS will assess whether your figures meet our technical requirements by comparing each figure against our figure specifications.

# Author comment: Author response to Decision Letter 1

**DOI:** 10.1371/journal.pone.0357053.r002

18 Jun 2026

Please see the attached.

Attachment Submitted filename: Response to Review.docx

# Peer review: Decision Letter 1

**DOI:** 10.1371/journal.pone.0357053.r003

**Authors:** Dina Aboelsoued

12 Jul 2026

Thank you for submitting your manuscript to PLOS ONE. After careful consideration, we feel that it has merit but does not fully meet PLOS ONE’s publication criteria as it currently stands. Therefore, we invite you to submit a revised version of the manuscript that addresses the points raised during the review process.

==============================

**ACADEMIC EDITOR:**

I am still a bit concerned about the calculation of change in tick species abundance between the years given the difference in sampling periods. Thank you to the authors for adding footnotes and discussion highlighting that differences are due to phenological differences. I suggest adding this note to the Methods to provide consistency and clarity for the readers and reduce overstating the significance of differences. Differences in abundance could also be related to different locations between the years (habitats, ecology, etc) and recommend adding this to the appropriate sections.

I also suggest, if possible, to add a figure (e.g., barplot) illustrating the percent responses from each year’s survey when questions were the same. Since the results are presented in separate sections for each survey, it is a bit difficult to compare presented percentages. A figure would provide a side-by-side visualization that readers can refer to, especially for the comparisons presented in the Discussion.

Line 39-43. The statements that tick attachment site remained consistent across body regions contradicts the findings in the Results (Lines 416-417) that tick attachment site varied significantly across years. Please reconcile or reword to clarify.

Line 50-51. Anaplasma phagocytophilum is the causative agent of anaplasmosis in humans too, not just equids. I suggest rewording to reduce potential confusion.

Line 62. I suggest adding sample sizes to contextualize these percentages. These are high values (66-100%), but sample sizes were relatively small (10-50).

Lines 92-94. Please re-word this statement. Only data from Year 2 was used to assess control measure efficacy while this statement makes it appear that Year 1 data were also included. As indicated in section headings, Year 1 was more aimed at developing confidence while Year 2 was more for assessing control.

Lines 104-107, 134-142. How long and for what period of time were participants recruited for each study year? Readers may be curious about recruitment details.

Line 223. Could you provide more details on this outlier horse? Was it in a particular habitat that was different than other horses? Was it the only horse sampled in a county? Was it one of the 8 that was in both years that you could compare counts across time? Were all the ticks submitted at one collection or were they collected over multiple checks?

Lines 280-282. Since this information is presented in the Methods, it is not necessary to repeat it here.

Line 380. How did the tick abundance and diversity compare between years for the 8 horses that participated in both study years? This could supplement the discussion and interpretation of differences between years for the full dataset.

Line 381-382. Please check your numbers. Removing 286 ticks from Year 1’s total (332) results in 46 adjusted ticks, not 51.

Line 451-452. An odds ratio of 0.49 translates to a 51% reduction in tick encounters. Please correct.

Line 484-485. Please revisit the description of “effect uncertain”. There appear to be three conditions that could fulfil the definition (ARR ≥15% OR NNT ≤7; 95% CI for OR includes 1.0; ARR includes 0) with the first and third contradictory.

Line 510-511. Please mention the difference in sampling duration between these years. Otherwise, incorrect conclusions can be drawn about tick population dynamics and risk in PA, especially for readers who only skim through a single section.

Lines 521-523. Another factor is the difference in sample sizes between the studies. The cited survey had >500 responses while the surveys in Year 1 and 2 each had an order of magnitude fewer responses (40-50).

Line 554. Please explain the statement of “lower-than-expected tick counts”. To which year is being referred? How does this relate to the frequent tick checks (daily or 2-6 times/week) mentioned in the previous lines?

Line 617. Since you did not statistically compare treated vs. untreated barriers, it is not appropriate to state one had “superior performance” compared to the other. Commenting on the certainty (i.e., 95% CI) of fitted estimates for each would be consistent with the presented analyses.

Lines 638-639. While the presented seasons align with known phenology of blacklegged tick lifestages, not all seasons were included in the study. Therefore, I suggest not presenting these as conclusions from the study itself.

Table 5. I suggest not using “prevalence” in the last column heading. This could be confused for infection prevalence instead of the percent of hoses with ticks that week. This column is also redundant with column three (“observations with tick presence”) and is not needed. Also, since presence and absence are mutually exclusive, please consider the value of presenting both in this table. Consider adding a column indicating the number of horses with observation each week.

==============================

Please submit your revised manuscript by Aug 26 2026 11:59PM. If you will need more time than this to complete your revisions, please reply to this message or contact the journal office at plosone@plos.org. When you're ready to submit your revision, log on to https://www.editorialmanager.com/pone/ and select the 'Submissions Needing Revision' folder to locate your manuscript file.

- A letter that responds to each point raised by the academic editor and reviewer(s). You should upload this letter as a separate file labeled 'Response to Reviewers'.
- A marked-up copy of your manuscript that highlights changes made to the original version. You should upload this as a separate file labeled 'Revised Manuscript with Track Changes'.
- An unmarked version of your revised paper without tracked changes. You should upload this as a separate file labeled 'Manuscript'.

If you would like to make changes to your financial disclosure, please include your updated statement in your cover letter. Guidelines for resubmitting your figure files are available below the reviewer comments at the end of this letter.

If applicable, we recommend that you deposit your laboratory protocols in protocols.io to enhance the reproducibility of your results. Protocols.io assigns your protocol its own identifier (DOI) so that it can be cited independently in the future. For instructions see: https://journals.plos.org/plosone/s/submission-guidelines#loc-laboratory-protocols. Additionally, PLOS ONE offers an option for publishing peer-reviewed Lab Protocol articles, which describe protocols hosted on protocols.io. Read more information on sharing protocols at https://plos.org/protocols?utm_medium=editorial-email&utm_source=authorletters&utm_campaign=protocols.

As the corresponding author, your ORCID iD is verified in the submission system and will appear in the published article. PLOS supports the use of ORCID, and we encourage all coauthors to register for an ORCID iD and use it as well. Please encourage your coauthors to verify their ORCID iD within the submission system before final acceptance, as unverified ORCID iDs will not appear in the published article. *Only*  the individual author can complete the verification step; PLOS staff *cannot*  verify ORCID iDs on behalf of authors.

We look forward to receiving your revised manuscript.

Kind regards,

Dina Aboelsoued, Ph.D.

Academic Editor

PLOS One

Journal Requirements:

If the reviewer comments include a recommendation to cite specific previously published works, please review and evaluate these publications to determine whether they are relevant and should be cited. There is no requirement to cite these works unless the editor has indicated otherwise.

Please review your reference list to ensure that it is complete and correct. If you have cited papers that have been retracted, please include the rationale for doing so in the manuscript text, or remove these references and replace them with relevant current references. Any changes to the reference list should be mentioned in the rebuttal letter that accompanies your revised manuscript. If you need to cite a retracted article, indicate the article’s retracted status in the References list and also include a citation and full reference for the retraction notice.

[Note: HTML markup is below. Please do not edit.]

[NOTE: If reviewer comments were submitted as an attachment file, they will be attached to this email and accessible via the submission site. Please log into your account, locate the manuscript record, and check for the action link "View Attachments". If this link does not appear, there are no attachment files.]

To ensure your figures meet our technical requirements, please review our figure guidelines: https://journals.plos.org/plosone/s/figures

You may also use PLOS’s free figure tool, NAAS, to help you prepare publication quality figures: https://journals.plos.org/plosone/s/figures#loc-tools-for-figure-preparation.

NAAS will assess whether your figures meet our technical requirements by comparing each figure against our figure specifications.

# Author comment: Author response to Decision Letter 2

**DOI:** 10.1371/journal.pone.0357053.r004

29 Jul 2026

March 10, 2026

Dina Aboelsoued, Ph.D.

Academic Editor

PLOS One

Dear Dr. Aboelsoued,

Thank you for the recent time and effort that you and the reviewers put into our manuscript, "Barriers to entry: A participatory science study of tick identification, monitoring, and physical barriers to reduce tick bites on horses,” manuscript #PONE-D-25-58073. The authors sincerely appreciate the time and comments provided by you and the reviewers. We have incorporated your suggestions into the final version of the paper and attached both a track changes and a clean version for further review. Please find responses to reviewer comments below.

Figure 2: The colour bars need labels. I know, from the figure caption, what the colours mean, but can't see the little numbers in the colour bars very well.

--> From what I can see on my end, Figure 2 is still not clearly labelled. I don't know if they did update the figure and I just can't see/access it? I will leave this up to the editor to make the final judgement on. The labels were provided on Figure 2. We double checked the submission and they were there.

Lines 553-554: "The detection of H. longicornis in 2023 collections, although numerically small, represents a significant finding..." -- This is important, and the finding of this tick is mentioned in several areas of the manuscript. What is the potential health significance of this tick to horses? Can it affect humans? Has it been found in Pennsylvania before, just not in Clinton? If so, where and when?

--> the authors answered these questions well in their direct response to the edit, but did not actually make the necessary modifications in the text itself. We have added the following sentence to the discussion “This parthenogenetic tick feeds on large ungulates and, although it has not been associated with transmission of equine pathogens, heavy infestations have caused blood loss, anemia, reduced productivity, and mortality in cattle, highlighting the potential impact of high tick burdens on large animal hosts [33].” As well as specifying that this tick had been previously found in Pennsylvania, this was just a new detection in Clinton County.

I am still a bit concerned about the calculation of change in tick species abundance between the years given the difference in sampling periods. Thank you to the authors for adding footnotes and discussion highlighting that differences are due to phenological differences. I suggest adding this note to the Methods to provide consistency and clarity for the readers and reduce overstating the significance of differences. Differences in abundance could also be related to different locations between the years (habitats, ecology, etc) and recommend adding this to the appropriate sections. This has been added to the Tick Species section of the methods “Because sampling periods differed between years, percent changes were used only as directional comparisons relative to the Year 1 baseline and should not be interpreted as standardized estimates of interannual change. Differences in species abundance may reflect seasonal tick phenology associated with the shorter, earlier sampling period in Year 2, as well as differences in sampling locations, habitat characteristics, and local ecological conditions between years.” And to the discussion we added “years reflects not only location and microecology differences, but likely primarily…”

I also suggest, if possible, to add a figure (e.g., barplot) illustrating the percent responses from each year’s survey when questions were the same. Since the results are presented in separate sections for each survey, it is a bit difficult to compare presented percentages. A figure would provide a side-by-side visualization that readers can refer to, especially for the comparisons presented in the Discussion. Only nine questions were identical across the two surveys. After reviewing these items, we determined that they did not provide a sufficiently comprehensive or informative basis for a meaningful comparative figure. We were concerned that presenting these limited items visually could overemphasize comparisons that were not central to the study’s objectives. Therefore, we elected not to add the proposed figure and instead retained the year-specific presentation of the results.

Line 39-43. The statements that tick attachment site remained consistent across body regions contradicts the findings in the Results (Lines 416-417) that tick attachment site varied significantly across years. Please reconcile or reword to clarify. This change has been reworded to “Tick attachment occurred across eight body locations and differed significantly between years. ” in the abstract.

Line 50-51. Anaplasma phagocytophilum is the causative agent of anaplasmosis in humans too, not just equids. I suggest rewording to reduce potential confusion. We eliminated “equine” in front of anaplasmosis to clarify.

Line 62. I suggest adding sample sizes to contextualize these percentages. These are high values (66-100%), but sample sizes were relatively small (10-50). Thank you for this suggestion. We have retained the original wording because the cited percentages are used only to illustrate geographic variation in reported seroprevalence, rather than to compare study precision or derive a pooled estimate.

Lines 92-94. Please re-word this statement. Only data from Year 2 was used to assess control measure efficacy while this statement makes it appear that Year 1 data were also included. As indicated in section headings, Year 1 was more aimed at developing confidence while Year 2 was more for assessing control. We moodified for clarity to “During Year 1, participants conducted systematic tick monitoring to develop proficiency with study protocols and characterize tick presence and attachment patterns on horses. During Year 2, participants implemented physical barrier interventions while continuing standardized tick surveillance, and only Year 2 data were used to evaluate intervention efficacy. Together, the two study phases generated information on tick species affecting horses in Pennsylvania, assessed the effectiveness of physical barriers for equine tick prevention, and demonstrated the feasibility of community-based research in equine health.”

Lines 104-107, 134-142. How long and for what period of time were participants recruited for each study year? Readers may be curious about recruitment details. We added the months that recruitment took place in the Materials and Methods section.

Line 223. Could you provide more details on this outlier horse? Was it in a particular habitat that was different than other horses? Was it the only horse sampled in a county? Was it one of the 8 that was in both years that you could compare counts across time? Were all the ticks submitted at one collection or were they collected over multiple checks? We reviewed the characteristics of this horse and considered whether additional description would improve interpretation of the results. Because this represented a single observation, we did not identify evidence that it reflected a unique habitat, county, sampling history, or other systematic factor relevant to the study objectives. Including these details would place undue emphasis on an isolated observation without changing the interpretation of the findings. Our intent was simply to acknowledge the presence of an unusually high tick count while focusing the analysis on population-level patterns. Therefore, we have elected not to expand this section further.

Lines 280-282. Since this information is presented in the Methods, it is not necessary to repeat it here. These sentences have been deleted as requested.

Line 380. How did the tick abundance and diversity compare between years for the 8 horses that participated in both study years? This could supplement the discussion and interpretation of differences between years for the full dataset. Thank you for this suggestion. We agree that repeated observations on the same horses could provide useful longitudinal insight in a larger study. However, only eight horses participated in both study years, representing a small, non-random subset of the study population. Given the limited sample size and the differences in environmental conditions and tick phenology between years, we do not believe that comparisons within these individuals would provide robust or generalizable conclusions. Because such analyses would be exploratory and potentially overinterpreted, we elected to focus on the population-level comparisons that align with the study objectives and design.

Line 381-382. Please check your numbers. Removing 286 ticks from Year 1’s total (332) results in 46 adjusted ticks, not 51.This adjustment has been made.

Line 451-452. An odds ratio of 0.49 translates to a 51% reduction in tick encounters. Please correct. This adjustment has been made.

Line 484-485. Please revisit the description of “effect uncertain”. There appear to be three conditions that could fulfil the definition (ARR ≥15% OR NNT ≤7; 95% CI for OR includes 1.0; ARR includes 0) with the first and third contradictory. Yes, this was a copy and paste error. This has been revised to be “Effect uncertain: the estimated effect did not meet the prespecified threshold for clinical importance (ARR <15% and NNT >7), or the confidence intervals indicated statistical imprecision by including 1.0 for the OR or 0 for the ARR.”

Line 510-511. Please mention the difference in sampling duration between these years. Otherwise, incorrect conclusions can be drawn about tick population dynamics and risk in PA, especially for readers who only skim through a single section. The sampling period was added “Sampling was conducted over seven weeks from April 18 to June 5 in 2022 and four weeks from April 3 to April 30 in 2023.”

Lines 521-523. Another factor is the difference in sample sizes between the studies. The cited survey had >500 responses while the surveys in Year 1 and 2 each had an order of magnitude fewer responses (40-50). Revised this section to say “The reason for the lower level of concern reported in Year 1 is unclear but may reflect differences in participants’ prior experience with tick-borne disease or the substantially smaller sample sizes in the current study, with approximately 40–50 respondents in each year.”

Line 554. Please explain the statement of “lower-than-expected tick counts”. To which year is being referred? How does this relate to the frequent tick checks (daily or 2-6 times/week) mentioned in the previous lines? This was referring to participant perceptions. This has been clarified to “Participants may have expected to collect more ticks than they ultimately found, and the lower-than-expected tick recovery may have contributed to this behavioral change by creating a false sense of security regarding the likelihood of tick bites.”

Line 617. Since you did not statistically compare treated vs. untreated barriers, it is not appropriate to state one had “superior performance” compared to the other. Commenting on the certainty (i.e., 95% CI) of fitted estimates for each would be consistent with the presented analyses. Thank you for this comment. We agree that our original wording implied a direct statistical comparison between untreated and permethrin-treated barriers that was not conducted. We have revised the statement to describe the fitted estimates and it now reads “The fitted estimates indicated that both untreated and permethrin-treated barriers reduced blacklegged tick attachment. These findings suggest that physical exclusion alone may contribute to protection against blacklegged ticks, as has been reported for stable flies.[”

Lines 638-639. While the presented seasons align with known phenology of blacklegged tick lifestages, not all seasons were included in the study. Therefore, I suggest not presenting these as conclusions from the study itself. The language of this section was modified to not be as specific with seasonality and instead include “seasonal risk.”

Table 5. I suggest not using “prevalence” in the last column heading. This could be confused for infection prevalence instead of the percent of hoses with ticks that week. This column is also redundant with column three (“observations with tick presence”) and is not needed. Also, since presence and absence are mutually exclusive, please consider the value of presenting both in this table. Consider adding a column indicating the number of horses with observation each week. We eliminated the weekly tick prevalence column and added the N of horses observed each week, as well as an explanatory footnote.

Again, it is with many thanks that I respond to your comments and concerns regarding this manuscript and offer my sincere appreciation for the time and effort you put forth in the editorial comments which, no doubt, will improve the document as a whole.

Sincerely,

Erika T. Machtinger, PhD

Pennsylvania State University

Attachment Submitted filename: Citizen Science Manuscript response to review_R2_07242026.docx

# Peer review: Decision Letter 2

**DOI:** 10.1371/journal.pone.0357053.r005

**Authors:** Dina Aboelsoued

12 Aug 2026

Barriers to entry: A participatory science study of tick identification, monitoring, and physical barriers to reduce tick bites on horses

PONE-D-25-58073R2

Dear Dr. Erika Machtinger

We’re pleased to inform you that your manuscript has been judged scientifically suitable for publication and will be formally accepted for publication once it meets all outstanding technical requirements.

Within one week, you’ll receive an e-mail detailing the required amendments. When these have been addressed, you’ll receive a formal acceptance letter and your manuscript will be scheduled for publication.

An invoice will be generated when your article is formally accepted. Please note, if your institution has a publishing partnership with PLOS and your article meets the relevant criteria, all or part of your publication costs will be covered. Please make sure your user information is up-to-date by logging into Editorial Manager at Editorial Manager® and clicking the ‘Update My Information' link at the top of the page. For questions related to billing, please contact billing support.

If your institution or institutions have a press office, please notify them about your upcoming paper to help maximize its impact. If they’ll be preparing press materials, please inform our press team as soon as possible -- no later than 48 hours after receiving the formal acceptance. Your manuscript will remain under strict press embargo until 2 pm Eastern Time on the date of publication. For more information, please contact onepress@plos.org.

Kind regards,

Dina Aboelsoued, Ph.D.

Academic Editor

PLOS One

Reviewers' comments:

Reviewer's Responses to Questions

**Comments to the Author**

Reviewer #2: All comments have been addressed

**********

2. Is the manuscript technically sound, and do the data support the conclusions?

Reviewer #2: (No Response)

**********

3. Has the statistical analysis been performed appropriately and rigorously?

Reviewer #2: (No Response)

**********

4. Have the authors made all data underlying the findings in their manuscript fully available?

The PLOS Data policy

Reviewer #2: (No Response)

**********

5. Is the manuscript presented in an intelligible fashion and written in standard English?

Reviewer #2: (No Response)

**********

Reviewer #2: (No Response)

**********

what does this mean?). If published, this will include your full peer review and any attached files.

If you choose “no”, your identity will remain anonymous but your review may still be made public.

**Do you want your identity to be public for this peer review?**  For information about this choice, including consent withdrawal, please see our Privacy Policy

Reviewer #2: No

**********

# Editor report: Acceptance letter

**DOI:** 10.1371/journal.pone.0357053.r006

**Authors:** Dina Aboelsoued

PONE-D-25-58073R2

PLOS One

Dear Dr. Machtinger,

I'm pleased to inform you that your manuscript has been deemed suitable for publication in PLOS One. Congratulations! Your manuscript is now being handed over to our production team.

At this stage, our production department will prepare your paper for publication. This includes ensuring the following:

* All references, tables, and figures are properly cited

* All relevant supporting information is included in the manuscript submission,

* There are no issues that prevent the paper from being properly typeset

You will receive further instructions from the production team, including instructions on how to review your proof when it is ready. Please keep in mind that we are working through a large volume of accepted articles, so please give us a few days to review your paper and let you know the next and final steps.

Lastly, if your institution or institutions have a press office, please let them know about your upcoming paper now to help maximize its impact. If they'll be preparing press materials, please inform our press team within the next 48 hours. Your manuscript will remain under strict press embargo until 2 pm Eastern Time on the date of publication. For more information, please contact onepress@plos.org.

You will receive an invoice from PLOS for your publication fee after your manuscript has reached the completed accept phase. If you receive an email requesting payment before acceptance or for any other service, this may be a phishing scheme. Learn how to identify phishing emails and protect your accounts at https://explore.plos.org/phishing.

If we can help with anything else, please email us at customercare@plos.org.

Thank you for submitting your work to PLOS One and supporting open access.

Kind regards,

PLOS One Editorial Office Staff

on behalf of

Dr. Dina Aboelsoued

Academic Editor

PLOS One
